# The nucleotide messenger (p)ppGpp is an anti-inducer of the purine synthesis transcription regulator PurR in *Bacillus*

**DOI:** 10.1093/nar/gkab1281

**Published:** 2021-12-30

**Authors:** Brent W Anderson, Maria A Schumacher, Jin Yang, Asan Turdiev, Husan Turdiev, Jeremy W Schroeder, Qixiang He, Vincent T Lee, Richard G Brennan, Jue D Wang

**Affiliations:** Department of Bacteriology, University of Wisconsin-Madison, Madison, WI 53706, USA; Department of Biochemistry, Duke University, Durham, NC 27710, USA; Department of Bacteriology, University of Wisconsin-Madison, Madison, WI 53706, USA; Department of Cell Biology & Molecular Genetics, University of Maryland, College Park, MD 20742, USA; Department of Cell Biology & Molecular Genetics, University of Maryland, College Park, MD 20742, USA; Department of Bacteriology, University of Wisconsin-Madison, Madison, WI 53706, USA; Department of Bacteriology, University of Wisconsin-Madison, Madison, WI 53706, USA; Department of Cell Biology & Molecular Genetics, University of Maryland, College Park, MD 20742, USA; Department of Biochemistry, Duke University, Durham, NC 27710, USA; Department of Bacteriology, University of Wisconsin-Madison, Madison, WI 53706, USA

## Abstract

The nucleotide messenger (p)ppGpp allows bacteria to adapt to fluctuating environments by reprogramming the transcriptome. Despite its well-recognized role in gene regulation, (p)ppGpp is only known to directly affect transcription in Proteobacteria by binding to the RNA polymerase. Here, we reveal a different mechanism of gene regulation by (p)ppGpp in Firmicutes: (p)ppGpp directly binds to the transcription factor PurR to downregulate purine biosynthesis gene expression upon amino acid starvation. We first identified PurR as a receptor of (p)ppGpp in *Bacillus anthracis*. A co-structure with *Bacillus subtilis* PurR reveals that (p)ppGpp binds to a PurR pocket reminiscent of the active site of phosphoribosyltransferase enzymes that has been repurposed to serve a purely regulatory role, where the effectors (p)ppGpp and PRPP compete to allosterically control transcription. PRPP inhibits PurR DNA binding to induce transcription of purine synthesis genes, whereas (p)ppGpp antagonizes PRPP to enhance PurR DNA binding and repress transcription. A (p)ppGpp-refractory *purR* mutant in *B. subtilis* fails to downregulate purine synthesis genes upon amino acid starvation. Our work establishes the precedent of (p)ppGpp as an effector of a classical transcription repressor and reveals the key function of (p)ppGpp in regulating nucleotide synthesis through gene regulation, from soil bacteria to pathogens.

## INTRODUCTION

Nucleotide second messengers allow organisms to rewire transcription to reprogram physiology and lifestyle for survival in changing environments. In bacteria, nucleotide second messengers, such as the cyclic nucleotides cAMP, c-di-AMP and c-di-GMP, regulate transcription by binding to transcription factors as effectors and allosterically affecting the transcription factor's binding to DNA regulatory elements (1–8). For example, cAMP, the key regulator of catabolite repression, was one of the first identified allosteric effectors of transcription, establishing the paradigm of gene regulation (9).

In contrast to cyclic nucleotides, another group of signaling nucleotides is the linear ‘alarmone’ nucleotides, including the ubiquitous nucleotide messengers ppGpp and pppGpp (collectively (p)ppGpp). (p)ppGpp is necessary for bacteria to adapt to environmental changes from nutrient availability to antibiotic assault (10–12). Since its discovery a half century ago, (p)ppGpp was best known for its profound role in gene regulation: downregulating the transcription of rRNA and tRNA operons and modulating global transcription in bacteria. (p)ppGpp binds RNA polymerase as well as the interface between RNA polymerase and the transcription factor DksA in Proteobacteria, and it binds the virulence regulator complex MglA-SspA in *Francisella tularensis* (13,14). However, unlike cyclic nucleotide second messengers, (p)ppGpp has not been known to act as an allosteric effector of a canonical promoter-binding transcription factor in bacteria.

In addition, (p)ppGpp has only been shown to regulate transcription directly in Proteobacteria (13–15). In other bacterial phyla, no transcription proteins have been shown to be directly regulated by (p)ppGpp. In Firmicutes, including the soil bacterium *Bacillus subtilis* and the pathogen *Bacillus anthracis*, (p)ppGpp affects the transcription of hundreds of genes, but the only known mechanism is indirect: via control of levels of GTP, which is the initiating nucleotide for ribosomal RNA genes and is an effector for the global regulator CodY (16–18). Therefore, the pervasiveness of (p)ppGpp's role in direct gene expression remains unclear.

Here, we report the first example of (p)ppGpp as an allosteric effector of a canonical transcription regulator, and the first example of transcription under direct control of (p)ppGpp in bacterial species beyond the Proteobacteria phylum. We found that (p)ppGpp directly regulates transcription in *Bacillus* by binding to the transcription regulator PurR and serving as its effector. PurR controls expression of *de novo* and salvage purine nucleotide biosynthesis genes in response to availability of external nucleobases (19,20). Here, we show that PurR also responds to amino acid starvation via (p)ppGpp induction, which strongly increases PurR repression of purine nucleotide biosynthesis genes. PurR proteins across Firmicutes comprise a DNA binding domain and a regulatory domain resembling phosphoribosyltransferase (PRT) enzymes. PRT enzymes, including HPRT and XPRT, are purine salvage enzymes that use the substrate 5-phosphoribosyl-1-pyrophosphate (PRPP) to produce purine nucleotides such as GMP, XMP and IMP. However, the PRT domain on PurR lacks enzymatic activity and serves a purely regulatory role by binding PRPP to de-repress transcription (20,21). Our structural and biochemical analyses reveal that (p)ppGpp also binds to the PRT regulatory domain and competes with PRPP to promote PurR binding to the promoter DNA, thus serving as a PurR anti-inducer. Our study highlights the conservation of the role of signaling nucleotides as allosteric effectors of gene regulation, not only by cyclic but also linear nucleotides.

## MATERIALS AND METHODS

### Cloning and strain construction

Primers, plasmids, and strains used in this study are listed in Supplementary Tables S3–S5. All primers were purchased from Integrated DNA Technologies (Coralville, IA, USA). For selection in *B. subtilis*, media was supplemented with antibiotics when required: MLS (erythromycin at 0.5 μg/ml and lincomycin at 12.5 μg/ml) and spectinomycin (80 μg/ml). For *E. coli*, carbenicillin was used at 100 μg/ml.

*B. subtilis purR* was cloned into pLIC-trPC-HMA. PurR variants were made by megaprimer site-directed mutagenesis of the plasmid (22). Inserts and mutants were confirmed by PCR amplification and Sanger sequencing.

*B. subtilis* NCIB 3610 *purR*::*ermHI* was constructed by amplifying the *purR*::*ermHI* genomic region from *B. subtilis* 168 *purR*::*ermHI* (BKE00470, *Bacillus* Genetic Stock Center). The PCR product was transformed into JDW2809, and transformants were selected for MLS resistance. Positive transformants were confirmed by PCR.

To construct *purR(D203A)* and *purR(Y102A)* mutants in *B. subtilis*, CRISPR-Cas9 plasmids were cloned with Golden Gate assembly of pJW557 (amplified with primers oJW2775/2821), the appropriate repair template (gene fragment from GeneWiz, South Plainfield, NJ, USA), and the appropriate guide RNA. Plasmids were transformed into JDW159 (*recA^+^ E. coli*) with selection on carbenicillin. Purified plasmid was transformed into JDW2809 and transformants were selected for spectinomycin resistance. Transformants were re-streaked twice on LB at 45°C to cure the plasmid. Transformants were patched on spectinomycin, and the *purR* region of spectinomycin-sensitive colonies was sequenced to confirm the mutation. To construct the *purR(Y102A/F205A)* and *purR(Y102A/K207A)* double mutants, CRISPR-Cas9 plasmids were constructed with repair templates for *purR(F205A)* and *purR(K207A*) with the same guide RNA as *purR(D203A)*. These plasmids were transformed into *purR(Y102A)*.

To construct P*_purE_*-sfGFP reporters, 500 bp DNA upstream of the start codon of *purE* in *B. subtilis* was amplified with primers oJW3438/3439 and cloned into pJW669 with Golden Gate assembly according to New England Biolabs (Ipswich, MA, USA) recommended protocol. The plasmid was transformed into JDW159 (*recA^+^ E. coli*) with selection on carbenicillin. Insert was confirmed by sequencing. Purified plasmid was transformed into JDW2809, JDW3970, JDW3975 and *B. subtilis* Δ*yjbM* Δ*ywaC*. Transformants were selected on spectinomycin and streaked twice on LB at 45°C to cure the plasmid. Spectinomycin-sensitive colonies were confirmed by PCR. (p)ppGpp^0^ with P*_purE_*-sfGFP was constructed by transforming a *relA*::*mls* PCR fragment amplified with primers oJW902/903 into *B. subtilis* Δ*yjbM* Δ*ywaC amyE*::P*_purE_*-sfGFP (11). Transformants were selected for MLS resistance and confirmed by PCR.

### Growth conditions

*B. subtilis* and *E. coli* strains were grown at 37°C unless otherwise noted. *E. coli* was grown in lysogeny broth (LB) or Terrific broth media. Media used to grow *B. subtilis* included LB and S7 defined minimal medium with 1% glucose (23). S7 was supplemented with 20 amino acids (18) or seven amino acids VILMTHE as needed. For experiments, a single colony of each strain was resuspended in 1X Spizizen salts (24) and spread on modified Spizizen minimal agar plates (1X Spizizen salts, 1% glucose, 2 mM MgCl_2_, 0.7 mM CaCl_2_, 50 mM MnCl_2_, 5 mM FeCl_3_, 1 mM ZnCl_2_, 2 mM thiamine, 1.5% agar and 0.1% glutamate). The minimal agar plates were supplemented with specific amino acids or 0.5% casamino acids where needed, especially in the case of amino acid auxotrophic (p)ppGpp^0^. The plates were incubated ∼16 h at 25–30°C and diluted to an OD_600_ ∼ 0.005 in indicated liquid media for liquid culture experiments.

Growth curves were performed with a Synergy 2 plate reader (BioTek, Winooski, VT, USA) to measure OD_600_ and GFP fluorescence (Ex_485_/Em_528_). To measure growth rate, cells were washed from overnight plates, resuspended in S7 medium with 20aa at OD_600_ 0.005, grown to OD_600_ ∼0.2, and diluted to OD_600_ 0.005 in a clear 96-well plate (Corning, Corning, NY, USA) for measuring OD_600_ in the plate reader. Doubling times were calculated using a custom Python script that measured the fastest growth rate in a one hour window. To measure GFP fluorescence from the P*_purE_*-sfGFP reporter, S7 medium with 20aa in a clear bottom black 96-well plate (Corning) was inoculated with part of a single colony of bacteria. Curves of OD_600_ and fluorescence were translated to a starting OD_600_ 0.075 for comparison across strains. To perform nutrient downshifts, cells were washed from overnight plates, resuspended in S7 medium with 20aa at OD_600_ 0.01, and grown to OD_600_ ∼0.2. The medium was then filtered out with Costar Spin-X centrifuge filters (Corning, 0.45 μm) by centrifuging at 5000 × *g* for one min. The cells were washed three times with S7 medium without any amino acids (except for glutamate), resuspended in the same medium, and diluted in a 96-well plate at OD_600_ 0.005 in both S7 medium with and without 20aa.

### ORFeome DRaCALA screen

*Bacillus anthracis* Gateway^®^ Clone Set containing plasmids bearing *B. anthracis* open reading frames was acquired from BEI Resources and used for Gateway cloning with the manufacturer's protocol (Invitrogen, Waltham, MA, USA) into overexpression vectors pVL791 (10xHis tag, ampicillin-resistant) and pVL847 (10xHis-MBP tag, gentamycin-resistant) and transformed into *Escherichia coli* BL21 lacI^q^ to produce two open reading frame proteome over-expression libraries (ORFeome library).

Cell lysate with overexpressed protein and purified protein were used for DRaCALA as described (25). 10 μl cell lysate or diluted, purified protein was mixed with 10 μl diluted [5′-α-^32^P]-pppGpp (∼0.2 nM) in a buffer containing 10 mM Tris pH 7.5, 100 mM NaCl and 5 mM MgCl_2_, incubated at room temperature for 10 min. ∼2 μl mixture was blotted onto nitrocellulose membrane (Amersham; GE Healthcare, Chicago, IL, USA) and allowed for diffusion and drying. The nitrocellulose membrane loaded with mixture was exposed on phosphor screen, which was scanned by a Typhoon FLA9000 scanner (GE Healthcare). Fraction of (p)ppGpp binding was analyzed as described (25).

### Protein purification

PurR proteins were expressed in *E. coli* BL21 T7 Express *lacI^q^*(New England Biolabs) from the pLIC-trPC-HMA plasmid with an N-terminal hexahistidine-maltose binding protein (HisMBP) tag. Seed cultures at OD_600_ ∼0.5 were diluted 1:50 into Terrific broth and grown to OD_600_ ∼1.5 at 37°C prior to induction with 1 mM IPTG for four hours. Cultures were centrifuged at 10 000 × *g* for 30 min, the pellet was washed with 25 ml 1× phosphate-buffered saline (PBS), and the pellet was stored at −80°C.

Pellets were resuspended in Lysis Buffer (25 mM Tris–HCl pH 7.5, 300 mM NaCl, 10 mM imidazole; ∼20 ml Lysis Buffer per 1 l cell pellet) with Pierce protease inhibitor tablets (Thermo Fisher Scientific, Waltham, MA, USA) and DNase I (MilliporeSigma, Burlington, MA, USA; 500 U/l cell pellet). Cells were lysed via French press and centrifuged at 30 000 × *g* for 30 min. The supernatant was filtered through a 0.45 μm filter. HisMBP-tagged PurR was loaded onto a HisTrap FF column (GE Healthcare) equilibrated with Lysis Buffer on an AktaPure FPLC (GE Healthcare). The column was washed with 15 column volumes (CV) of Lysis Buffer with 5% Elution Buffer (25 mM Tris–HCl pH 7.5, 300 mM NaCl, 500 mM imidazole). Protein was eluted in a gradient elution from 5 to 50% Elution Buffer. HisMBP-tagged PurR was dialyzed with tobacco etch virus (TEV) protease overnight in 25 mM Tris–HCl pH 8, 200 mM NaCl, 1 mM dithiothreitol (DTT), 0.5 mM ethylenediaminetetraacetic acid (EDTA) and 10 mM (NH_4_)_2_SO_4_. Following cleavage of the HisMBP tag with TEV protease, dialyzed protein was transferred into Lysis Buffer with a HiPrep 26/10 desalting column (GE Healthcare) and passed over a HisTrap FF column. Flowthrough was collected as untagged PurR. Untagged PurR was further purified via HiPrep 26/60 Sephacryl S-200 gel filtration (GE Healthcare) equilibrated in 20 mM HEPES pH 8, 300 mM NaCl and 50 mM (NH_4_)_2_SO_4_. The peak corresponding to PurR was collected, concentrated, and concentration was measured with the Bradford assay. Typical concentrations were 10–20 mg/ml and yields were ∼3–4 mg PurR/l culture. Protein was flash-frozen in liquid nitrogen and stored at −80°C.

For small scale purification of HisMBP-tagged PurR, protein was expressed the same as above in a 5 ml volume. The cell pellet was stored at −20°C until used. Cells were resuspended in one ml lysis buffer (20 mM sodium phosphate pH 8, 500 mM NaCl, and 10 mM imidazole). Cells were incubated with 1.6 mg lysozyme (MilliporeSigma) and 360 U Benzonase endonuclease (MilliporeSigma) for about one hour on ice for lysis. Lysate was centrifuged at 20 000 × *g* for 15 min. Supernatant was incubated with His Mag Sepharose beads (equilibrated with lysis buffer according to manufacturer's instructions; GE Healthcare) for 30 min at 4°C rotating end-over-end. Beads were washed three times with 500 μl wash buffer (same as lysis buffer but with 40 mM imidazole). Protein was eluted three times with 250 μl elution buffer (same as lysis buffer but with 500 mM imidazole). Eluted protein was dialyzed into 20 mM HEPES pH 8, 300 mM NaCl, 20 mM (NH_4_)_2_SO_4_, concentration was measured with *A*_280_ and extinction coefficient, and flash frozen with liquid nitrogen for storage at -80°C.

### DRaCALA

[5′-α-^32^P] pppGpp and ppGpp were purified as previously described (26,27). For PurR DRaCALA, protein in storage buffer was diluted to the appropriate concentration in 20 mM HEPES pH 8, 100 mM NaCl. ^32^P-labeled pppGpp or ppGpp was added in a final dilution of ∼1:50 from its stock. The protein and ligand were incubated for 10 min and spotted onto Protran BA85 nitrocellulose (0.45 μm, GE Healthcare) with a replicator tool (VP 408FP6S2; V&P Scientific, Inc., San Diego, CA, USA). After the spots dried, the nitrocellulose was exposed to a phosphor screen for at least three hours and scanned with a Typhoon FLA9000 phosphorimager. Images were analyzed with ImageJ and fraction bound ^32^P-labeled (p)ppGpp was calculated as described (25). For ppGpp-PRPP competition assays, non-radiolabeled PRPP was first incubated with PurR (diluted as described above) for about two minutes before radiolabeled ppGpp was added. The reaction was then incubated for 10 min before being processed as described above. Data were analyzed with Prism v7 (GraphPad Software, San Diego, CA, USA), and binding curves were obtained by fitting the data to the equation: *Y* = (*B*_max_ × *X^h^*) / (*K*_d_^*h*^ + *X^h^*), where *h* represents the Hill cooperativity coefficient. ppGpp-PRPP competition data were fit to a four-parameter logistic regression model in Prism. All DRaCALA reactions were performed in technical triplicate (separate reactions from the same protein preparation) and variance was determined as standard error of the mean.

### X-ray crystallography

To obtain PurR-ppGpp crystals, the purified PurR protein was concentrated to 20 mg/ml and ppGpp was added to a final concentration of 1 mM. Crystal screens were performed using the hanging drop vapor diffusion method. The best crystals were obtained by mixing this complex 1:1 with a crystallization reservoir consisting of 0.1 M 2-(*N*-morpholino)ethanesulfonic acid (MES) pH 6.5 and 18% PEG 1500. The crystals grew at room temperature and took from 5 days to 1 week to reach optimal size. The crystals take the triclinic space group P1 and diffracted beyond 2.5 Å on synchrotron sources. The crystals were cryo-protected by dipping the crystals in a 1 μl drop consisting of the crystallization reagent supplemented with 15% glycerol for 2 s. The crystal was then placed directly in the cryo-stream. X-ray intensity data were collected at the Advanced Light Source (ALS) beamline 8.3.1. The data were integrated in MOSFLM and scaled using SCALA (28). Phaser in CCP4 was used to solve the structure by Molecular replacement using a dimer of the 1P4A *B. subtilis* PurR structure with the ligand removed. Phaser readily located the three PurR dimers in the crystallographic asymmetric unit. Phenix (29) was then employed to perform refinement. After several rounds of refinement and refitting and optimizing of the model, density was evident for ppGpp molecules in four of the six PurR subunits; there was only weak density for the ppGpp molecules in the other two subunits. After fitting the ppGpp molecules, refinement in Phenix commenced and in the final stages of refinement ordered solvent molecules were added. The final model has *R*_work_/*R*_free_ values of 19.4%/23.9% to 2.45 Å resolution and validation in Molprobity placed the structure in the top 98% of structures solved to similar resolutions.

### Electrophoretic mobility shift assay (EMSA)

Gel shift assays were performed with untagged *B. subtilis* PurR, ppGpp, PRPP, and a 221 bp FAM-labeled DNA probe or a 202 bp unlabeled DNA probe. The 221 bp probe was amplified from upstream of the *pur* operon with oJW4028/4029, and the 202 bp probe was amplified with oJW1285/1286. DNA concentration was measured with Quant-IT (Thermo Fisher Scientific). Protein was diluted from frozen stock to appropriate concentrations in 10 mM HEPES pH 8.0, 100 mM KCl and 10% glycerol. For reactions with the FAM-labeled probe, the reaction contained 5 nM FAM-labeled DNA probe, 100 nM salmon sperm nonspecific DNA (Invitrogen), 10 mM HEPES pH 7.5, 200 mM KCl, 10% glycerol, 0.2 mM EDTA, 5 mM MgCl_2_, 1 mM DTT and 0.5 mg/ml bovine serum albumin (BSA; protein standard, Bio-Rad, Hercules, CA, USA). For reactions with the unlabeled DNA probe, the conditions were the same except 50 mM KCl, no nonspecific DNA and 1 nM DNA probe. Twenty microliter reactions were prepared from a 5× buffer, 10× protein stock, 10× DNA probe stock and 10× ppGpp or PRPP stock. Reactions were mixed by pipetting and incubated at room temperature (∼23°C) for 30 min. A 6% TBE polyacrylamide gel (Novex, Thermo Fisher Scientific) was pre-run at 10 V for 10 min at 4°C. Ten microliters of the sample were loaded and the gel was run at 100 V for ∼100 min at 4°C. For the unlabeled 202 bp probe, gels were stained with 1X SYBR Gold (Thermo Fisher Scientific) at room temperature for 30 min and imaged on a Typhoon FLA9000. For the FAM-labeled probe, gels were imaged on a Typhoon FLA9000 with FAM detection settings.

### LC–MS quantification of PRPP and other nucleotides

JDW2809 was grown in S7 defined media with 1% glucose and 0.1% potassium glutamate to OD_600_ ∼0.3. 5 ml culture was sampled before and after 0.5 mg/ml arginine hydroxamate treatment at defined time points. Sampled cultures were extracted and analyzed by LC–MS as described in previous publication (30). Concentration of PRPP was estimated as follows: PRPP concentration is 2.50 nmol/(mg dry weight) during exponential growth in minimal media (31). Given the dry weight/wet weight ratio of *B. subtilis* of 51.4% (32), the PRPP concentration is 2.50 × 51.4% = 1.29 nmol/(mg wet weight). Given the cell density is very close to water (1 g/ml), the PRPP concentration is 1.29 mM. Finally, we calculated PRPP concentration after arginine hydroxamate treatment for 30 min to be ∼0.7 mM by comparing the relative normalized ion counts to the untreated samples.

### Production of radiolabeled PurBox DNA probe

To construct the DNA probe for DNase I footprinting, the fragment containing PurBoxes was amplified by PCR using oligos oJW4280/oJW4281. This fragment was cloned into pUC18 vector to construct pJW747 by restriction digestion and ligation with HindIII, BamHI and T4 DNA ligase (New England Biolabs). pJW747 was processed as follows to produce radiolabeled PurBox DNA probe: 7.5 μg pJW747 was firstly digested by 20 U BamHI at 37°C for 30 min. The product was phenol extracted and treated by 10 U Quick CIP (New England Biolabs) at 37°C for 30 min. The product was heated at 80°C for 10 min to deactivate Quick CIP and incubated with 10 U T4 polynucleotide kinase (New England Biolabs) and 120 μCi [^32^P-α]-ATP (PerkinElmer, Waltham, MA, USA) to label the DNA. The product was heated at 65°C for 10 min to deactivate T4 polynucleotide kinase and treated by 20 U HindIII at 37°C for 30 min. The product was purified by phenol extraction and purified as described before (33).

### DNase I footprinting assay

DNase I footprinting reaction was performed as described before (33) with modifications. Footprinting was performed in 10 mM HEPES pH 7.5, 50 mM KCl, 10% glycerol, 1 mM EDTA, 5 mM MgCl_2_, 5 mM DTT, 2.5 mg/ml BSA and 5 ng/uL salmon sperm non-specific DNA. A 25 μl reaction mix with radiolabeled PurBox DNA probe was mixed with designated amounts of PurR, ppGpp and PRPP and incubated at room temperature for 1 h. The mixture was then treated with 3.2 μg/ml DNase I (Roche, Basel, Switzerland) for 40 s, and the reaction was stopped by adding 300 mM sodium acetate and 10 mM EDTA. The mix was purified by phenol extraction and dissolved in 5 μl gel loading buffer (8 M urea, 0.5× Tris–borate–EDTA buffer (TBE), 0.05% (w/v) bromophenol blue). The A + G ladder for footprinting was prepared as described before (33 ). DNA footprint gel electrophoresis was performed as described before (33). After electrophoresis, the dried gel was exposed to a phosphor screen for 48 h, and the screen was scanned on a Typhoon FLA9000 phosphorimager. The figure was analyzed by ImageJ for quantification of band intensities.

### Western blot analysis

To test for α-PurR antibody activity, cultures were obtained by inoculating LB with single colonies of wild-type (JDW2809) and *purR::ermHI B. subtilis* (JDW3359). Ten mL of culture were pelleted at 4500 × *g* for 20 min and washed with one mL of 50 mM Tris pH 7.5 and 300 mM NaCl. The pellet was resuspended in 100 μl of lysis buffer (10 mM Tris pH 7.5, 10 mM EDTA, 0.1 mg/ml lysozyme, 0.5 mM PMSF). Cells were lysed by incubation at 37°C for 30 min. An equal volume of 2× Laemmli buffer (Bio-Rad) was added and cells were incubated at 95°C for 10 min. The solution was centrifuged at maximum speed and the supernatant was transferred to a fresh tube. Ten microliters of the cell lysate were loaded onto a 12.5% polyacrylamide gel for western blot analysis. The protein was transferred to BA85 Protran nitrocellulose (GE Healthcare) with a semi-dry transfer apparatus (Bio-Rad) at 15 V for 30 min. The nitrocellulose was blocked with 1× PBST (1× PBS, 2% milk, 0.1% Tween-20) for four hours. The membrane was incubated overnight at 4°C with 10 ml PBST and 20 μl α-PurR rabbit serum (polyclonal, 1:500 dilution, raised against untagged purified *B. subtilis* PurR by Covance-Labcorp, Princeton, NJ, USA). The membrane was washed with 1× PBS before incubating for one hour with 10 ml 1× PBS and 0.5 μl secondary antibody (goat α-rabbit Alexa Fluor 647, Invitrogen). The membrane was washed three times with PBST and three times with PBS prior to imaging on a Odyssey CLx (LI-COR, Lincoln, NE, USA).

### ChIP-seq

The PurR ChIP protocol was adapted from previously published ChIP protocols (34,35). Cultures for ChIP-seq were grown in minimal medium supplemented with the amino acids VILMTHE. For ChIP-seq with nucleobases, the medium was supplemented with 1× ACGU (Teknova, Hollister, CA, USA). For sample collection, cells were grown from a starting OD_600_ ∼0.01. Cells were grown to an OD_600_ ∼0.5 at 37°C and 250 RPM. For RHX treatment, an untreated sample was collected by adding ∼50 ml culture into 1.4 ml 37% formaldehyde in a 50-ml conical. The conical was shaken at 37°C for 7–8 min prior to placing on ice. The remaining culture was treated with 0.5 mg/ml RHX and another ∼50 ml was crosslinked 10 min after RHX addition. Crosslinked samples were held on ice for at least 30 min, centrifuged at 5000 × *g* for 15 min, washed twice with ice-cold 25 ml 1× PBS, and resuspended in 1 ml ice-cold solution A (20% sucrose, 10 mM Tris pH 8, 10 mM EDTA, 50 mM NaCl). After resuspension in Solution A, samples were split into two equal volume microcentrifuge tubes and stored at –80°C. Crosslinked samples were collected from three biological replicates, each grown from a single colony, of wild-type and (p)ppGpp^0^*B. subtilis*. The following ChIP-seq protocol was used on each biological replicate separately.

For chromatin immunoprecipitation, one tube of each sample was thawed and treated with 1 mg/ml lysozyme (MilliporeSigma) at 37°C for 30 min. Lysed sample was diluted with 600 μl 2× IP buffer (100 mM Tris pH 7, 300 mM NaCl, 10 mM EDTA, 2% Triton X-100) and 0.5 mM AEBSF and divided into two tubes ∼550 μl each. Samples were sonicated with a Misonix S-4000 (Misonix, Inc., Farmingdale, NY, USA) with the cup horn attachment at amplitude 80 for 10 s ON, 15 s OFF, and a total ON time of 8–10 min. Like samples were recombined and 100 μl of sonicated sample was removed as the input sample. To each sonicated sample, 10 μl of polyclonal α-*B. subtilis* PurR serum (serum from terminal bleed of rabbit WI606 raised by Covance) was added. Antibody and lysate were incubated end-over-end overnight at 4°C. Antibody/protein mixture was added to 50 μl Dynabeads Protein A (Thermo Fisher Scientific) and incubated end-over-end for 1 h at 4°C. Beads were washed four times with lysis buffer 150 (50 mM HEPES–KOH pH 7.5, 150 mM NaCl, 1 mM EDTA, 1% Triton X-100, 0.1% SDS, 0.1% sodium deoxycholate) and two times with 10 mM Tris pH 7.5. See (34) for details on washes. Beads were resuspended in a 100 μl blunt enzyme mix reaction (Quick Blunting Kit, New England Biolabs) with half the enzyme concentration of the manufacturer's protocol. The blunting and 5′ phosphorylation reaction occurred at 24°C for 30 min with gentle rotation. Beads were then washed twice with lysis buffer 150 and twice with 10 mM Tris pH 8. A-tails were added to the 3′ ends of the DNA by incubating the beads with Klenow fragment (exo-) (New England Biolabs) in a 100 μl reaction. Following 30 min incubation at 37°C, beads were washed twice with lysis buffer 150 and twice with 10 mM Tris pH 7.5. Annealed dual-index Illumina adapters were ligated onto DNA by resuspending the beads in a 100 μl reaction containing Quick Ligase (New England Biolabs) and 0.1 μM annealed i5/i7 adapter. The ligation reaction was incubated for 15 min at 24°C. The beads were washed twice with lysis buffer 150, once with lysis buffer 500 (same composition as lysis buffer 150 except 500 mM NaCl), once with ChIP wash buffer (10 mM Tris pH 8, 250 mM LiCl, 1 mM EDTA, 0.5% sodium deoxycholate, 0.5% Nonidet-P40 (substitute) (Dot Scientific, Burton, MI, USA)), and once with Tris-EDTA buffer. Beads were resuspended in 48 μl elution buffer (50 mM Tris pH 8, 1 mM EDTA, 1% SDS) and 1.6 U Proteinase K (New England Biolabs), incubated at 55°C for 1 h and then 65°C overnight. Supernatant was transferred to a new tube and an additional 19 μl elution buffer and 0.8 U Proteinase K were added to the beads. Following incubation at 55°C for 1 h, reverse-crosslinked samples were combined and the DNA was purified using solid phase reverse immobilization (SPRI) Sera-Mag SpeedBeads (1.6:1 ratio, GE Healthcare). The final library was eluted with 22 μl ddH_2_O.

Input samples were processed by first adding 0.5 mg/ml RNase A (Thermo Fisher Scientific) and incubating at 37°C for at least 1 h. Then, 48 μl elution buffer and 1.6 U Proteinase K were added, incubated at 55°C for one hour, and then at 65°C overnight to reverse crosslinks. The DNA was isolated with a 1.8:1 SPRI cleanup and the concentration was quantified with Quant-iT (Thermo Fisher Scientific). Moving forward, 100 ng input DNA was processed with reactions similar to the ChIP samples, except the reaction volumes were 50 μl. Following blunting/5′ phosphorylation and A-tailing, the DNA was purified with a 1.8:1 SPRI cleanup. Following ligation, the DNA was purified with two successive 1:1 or 1.2:1 SPRI cleanups to eliminate adapter carryover. The final library was eluted with 22 μl ddH_2_O.

Quantitative PCR was used to determine the number of cycles for library amplification. In a 20 μl reaction, 2 μl of each sample DNA was combined with 0.2 μM each of oJW3536 and oJW3537, 1X EvaGreen dye (Biotium, Fremont, CA, USA), and KAPA high-fidelity low-bias polymerase (Roche). Samples were denatured in a Bio-Rad CFX qPCR thermocycler at 95°C for 2 min followed by 40 cycles of 95°C for 10 s, 55°C for 10 s, and 72°C for 30 s. The cycle quantification (C_q_) value provided by the program was used as the number of cycles for full library amplification. The remaining 20 μl of library was amplified with KAPA polymerase and the primers and concentrations listed above. The reactions were denatured at 98°C for 30 s, cycled the C_q_ value through 98°C for 10 s, 55°C for 15 s, and 72°C for 30 s before a final extension of 72°C for 5 min. Final amplified libraries were purified with a 1.6:1 SPRI cleanup and eluted with ddH_2_O.

### ChIP-seq data analysis

Libraries were sequenced on the Illumina NextSeq platform at the University of Michigan Advanced Genomics Core. The analysis pipeline and scripts are available at https://github.com/bwanderson3/2021_PurR_ChIP_analysis.git. Briefly, quality control was determined with FastQC (https://www.bioinformatics.babraham.ac.uk/projects/fastqc/). Adapters were removed with CutAdapt with arguments –quality-base = 33 -a AGATCGGAAGAGC -a CGGAAGAGCACAC -a GGGGGGGGGGGGGGG -n 3 -m 20 –match-read-wildcards -o. Using bowtie2 and arguments -U -q –end-to-end –very-sensitive -p 6 –phred33, wild-type *B. subtilis* reads were aligned to the *B. subtilis* NCIB 3610 genome (CP020102) from NCBI Reference Sequence Database. *B. subtilis* (p)ppGpp^0^ reads were aligned to the NCIB 3610 genome with the pBS32 megaplasmid (CP020102 concatenated with CP020103). Using a custom python script, coverage at a 10 bp resolution was bootstrapped and the median coverage of 100 bootstraps was used for downstream analysis. Coverage was smoothed with a custom python script. Enrichment was calculated as the median counts per million in a ChIP sample divided by the median counts per million in its corresponding input sample for each 10 bp window. The input sequencing dataset for the first replicate was low quality, so the input datasets from the same strain background and treatment conditions of the third replicate were used to calculate enrichment for the first replicate. For plotting enrichment, coverage was smoothed to 50 bp windows and enrichment was calculated again as above.

To calculate the effect of (p)ppGpp on PurR occupancy genome-wide, bootstrapped coverage at a 10 bp resolution that was determined in the previous paragraph was used. The effect of (p)ppGpp was determined by calculating: (enrichment_post_RHX_wildtype/enrichment_pre_RHX_wildtype)/(enrichment_post_RHX_ppGpp^0^/enrichment_pre_RHX_ppGpp^0^). This yielded 100 bootstraps for each biological replicate (replicate 1 for wild type was paired with replicate 1 for (p)ppGpp^0^ in the calculation). The median bootstrap value was determined from the 100 bootstraps for each biological replicate. Means and standard deviations were calculated from the medians of each biological replicate.

### PurR phylogenetic analysis

GenBank files and genome sequences were downloaded from NCBI Assembly for 106 complete reference bacterial genomes. To build the phylogenetic tree, 16S rRNA sequences were extracted from the GenBank files with a custom python script, and three eukaryotic 18S rRNA sequences were included as an outgroup. The rRNA sequences were aligned in MEGA X with ClustalW (36). The best model for tree construction was chosen as General Time Reversible with gamma distributed categories and invariant sites, which had the lowest AIC by MEGA X’s model test software (37). The tree was constructed according to this model in MEGA X with extensive grafting and subtree pruning and 750 bootstrap replicates. The tree was modified and annotated in R using the ggtree package (38).

Standalone BLAST+ from NCBI was used to determine which reference genomes contained a *B. subtilis* or *B. anthracis* PurR homolog. A BLAST database was created from the FASTA files of the same reference genome sequences used to generate the phylogenetic tree. The 73 N-terminal residues of *B. subtilis* PurR were BLASTed against this database with tblastn and a default E value of 10. This domain is unique to PurR according to Pfam (PF09182). The complete PurR sequence was not used due to homology of the PRT domain with PRT enzymes.

### Phylogenetic analysis of the PurR (p)ppGpp binding pocket

Conservation analysis of the protein and binding pocket was conducted on 938 PurR homologs in the Pfam family PF09182. Amino acid sequences were downloaded from UniProt, and proteins were aligned with MUSCLE in MEGA X. A frequency logo was created from aligned (p)ppGpp binding residues using WebLogo (UC Berkeley; https://weblogo.berkeley.edu/logo.cgi). ConSurf analysis was conducted using the ConSurf server (http://consurf.tau.ac.il/) with conservation score determined by Bayesian inference (39,40). Conservation scores were mapped onto PDB ID 1O57.

## RESULTS

### Screening a *B. anthracis* ORF library identifies the purine transcription regulator PurR as a (p)ppGpp target

**Figure 1.**
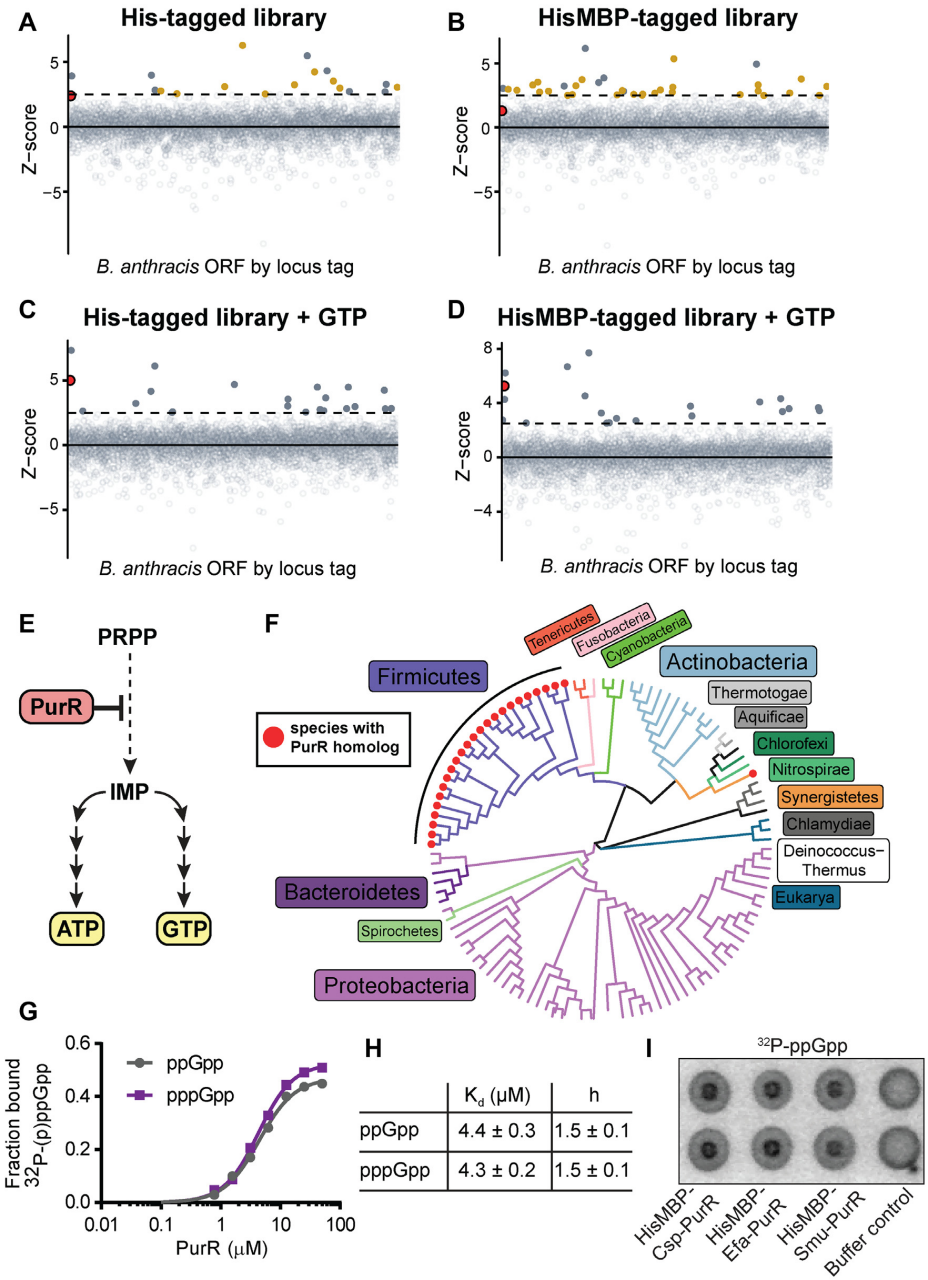
DRaCALA screen identifies the transcription factor PurR as a (p)ppGpp target in Firmicutes. (**A**, **B**) *Z*-scores of ^32^P-pppGpp binding to an open reading frame (ORF) library from *B. anthracis*. His indicates hexahistidine-tagged ORFs, and HisMBP indicates hexahistidine maltose binding protein tagged ORFs. ORFs with Z-scores greater than 2.5σ are filled. The dashed horizontal line is at 2.5σ. The red circle is the PurR ORF. Yellow symbols indicate ORFs that were hits without GTP competition but were not hits with GTP (see C, D). These data were previously reported in Yang *et al.* (2020). (**C**, **D**) DRaCALA of ORF libraries from (A) and (B) with 100 μM non-radioactive GTP as a competitor to reveal (p)ppGpp-specific targets. ^32^P-pppGpp is at ∼0.2 nM. (**E**) Schematic of PurR regulation of *de novo* ATP and GTP synthesis. (**F**) Cladogram constructed from 106 bacterial 16S rRNA and three eukaryotic 18S rRNA sequences. Tree branches colored according to the phylum. A red dot on the branch tip indicates that the species contains a *B. subtilis* PurR homolog. (**G**) Binding curve between purified untagged *B. subtilis* PurR and ^32^P-ppGpp (gray) and ^32^P-pppGpp (purple) obtained with DRaCALA. Error bars represent SEM of technical triplicate. Error bars are not visible when they are smaller than the height of the symbols. (**H**) Binding parameters from (**G**). mean ± standard error. *h* = Hill coefficient. (**I**) DRaCALA of ^32^P-ppGpp binding to 30 μM purified HisMBP-tagged PurR proteins from *Clostridium sporogenes* (Csp), *Enterococcus faecalis* (Efa), and *Streptococcus mutans* (Smu). Control is protein storage buffer.

(p)ppGpp has been shown to bind to the RNA polymerase core enzyme in Proteobacteria (14,41,42). In contrast, in Firmicutes (p)ppGpp has not been shown to regulate transcription directly (43), despite regulating many targets, including nucleotide synthesis enzymes, ribosome associated GTPases, and DNA replication enzymes (11,26,27,30,44,45). To uncover potential (p)ppGpp regulated transcriptional targets, we conducted a proteome based screen for proteins that directly bind (p)ppGpp in the pathogenic Firmicute *Bacillus anthracis* (30). We screened the 5341 open reading frame (ORF) library from *B. anthracis*, after placing each ORF into two expression constructs, one expressing the ORF with an N-terminal histidine (His) tag and the other with an N-terminal histidine maltose binding protein (HisMBP) tag. Each ORF in the His-tagged and HisMBP-tagged library was overexpressed and binding to ^32^P-pppGpp was assayed using differential radial capillary action of ligand assay (DRaCALA). The fraction of ^32^P-pppGpp bound to protein in each lysate was normalized as a *Z*-score for each plate (the number of standard deviations from the mean fraction ^32^P-pppGpp bound for each plate) to reduce the influence of plate-to-plate variation. We used a strict Z-score cutoff of 2.5 to identify proteins very likely to be (p)ppGpp targets (Figure 1A and B). This yielded known and new (p)ppGpp targets, as listed in Supplementary Table S1 and reported in (30).

Since (p)ppGpp binds many proteins in cell lysates, we sought to reduce nonspecific background binding by performing the same screen with a small amount (100 μM GTP) of non-radiolabeled GTP as a competitor (Figure 1C and D). Many (p)ppGpp binding proteins also bind GTP, so the non-radiolabeled GTP competitor reduces the *Z*-score of GTP binding proteins among others, leading to many hits losing significance in the presence of GTP (these are colored yellow in Figure 1A and B; Supplementary Table S1). This approach enriches for hits that specifically bind (p)ppGpp without competing with GTP. Strikingly, the purine synthesis transcription regulator PurR (BA0044) which had a *Z*-score slightly below the cutoff of 2.5 in the previous screens (Figure 1A and B), emerged as one of the strongest hits in the screens with GTP, only behind the known targets HPRT, XPRT, and YjbM (SasB) (Figure 1C and D, and Supplementary Table S1).

PurR is a transcription regulator in bacteria with the major role of repressing the transcription of genes responsible for *de novo* synthesis of IMP, the common precursor to the purine nucleotides ATP and GTP (Figure 1E) (20,46–48). PurR is known to be regulated by the availability of purine bases for salvage, thus is a critical regulator in ensuring balanced and efficient synthesis of purine nucleotides (20,49,50).

Homologs of the *B. anthracis* PurR are found almost exclusively in the bacterial phylum Firmicutes (Figure 1F). PurR proteins from other bacterial phyla, such as Proteobacteria, are structurally unrelated (51–53). To perform an in-depth study of the PurR-(p)ppGpp interaction, we turned to PurR from *B. subtilis*, a close relative of *B. anthracis*, since its PurR has been extensively characterized and the PurR homologs are similar (64% identical) (20). We purified untagged *B. subtilis* PurR and tested for binding to ^32^P-ppGpp and ^32^P-pppGpp with DRaCALA (Figure 1G). Both ppGpp and pppGpp interacted with *B. subtilis* PurR similarly, with binding curves best fit with a *K*_d_ ∼ 4.4 μM and a Hill cooperativity coefficient of 1.5 (Figure 1G and H). Given that the physiological concentration of (p)ppGpp in *Bacillus* can reach up to mM during amino acid starvation (11), this interaction is likely to be relevant *in vivo* and represents a potential new regulation of gene expression of the PurR regulon by amino acid starvation.

Next, we tested whether (p)ppGpp interaction extends to PurR homologs in other Firmicutes beyond *B. anthracis* and *B. subtilis*, using purified PurR from the soil bacterium *Clostridium sporogenes*, the opportunistic pathogen *Enterococcus faecalis*, and the oral pathogen *Streptococcus mutans*. In all cases, we detected significant binding to ^32^P-ppGpp (Figure 1I). Altogether, our screen has identified a transcription factor in Firmicutes that (p)ppGpp binds tightly and specifically. (p)ppGpp binding to PurR suggests that it can act as an effector for the transcription factor.

### (p)ppGpp binds to a conserved pocket on the effector binding domain of PurR

**Figure 2.**
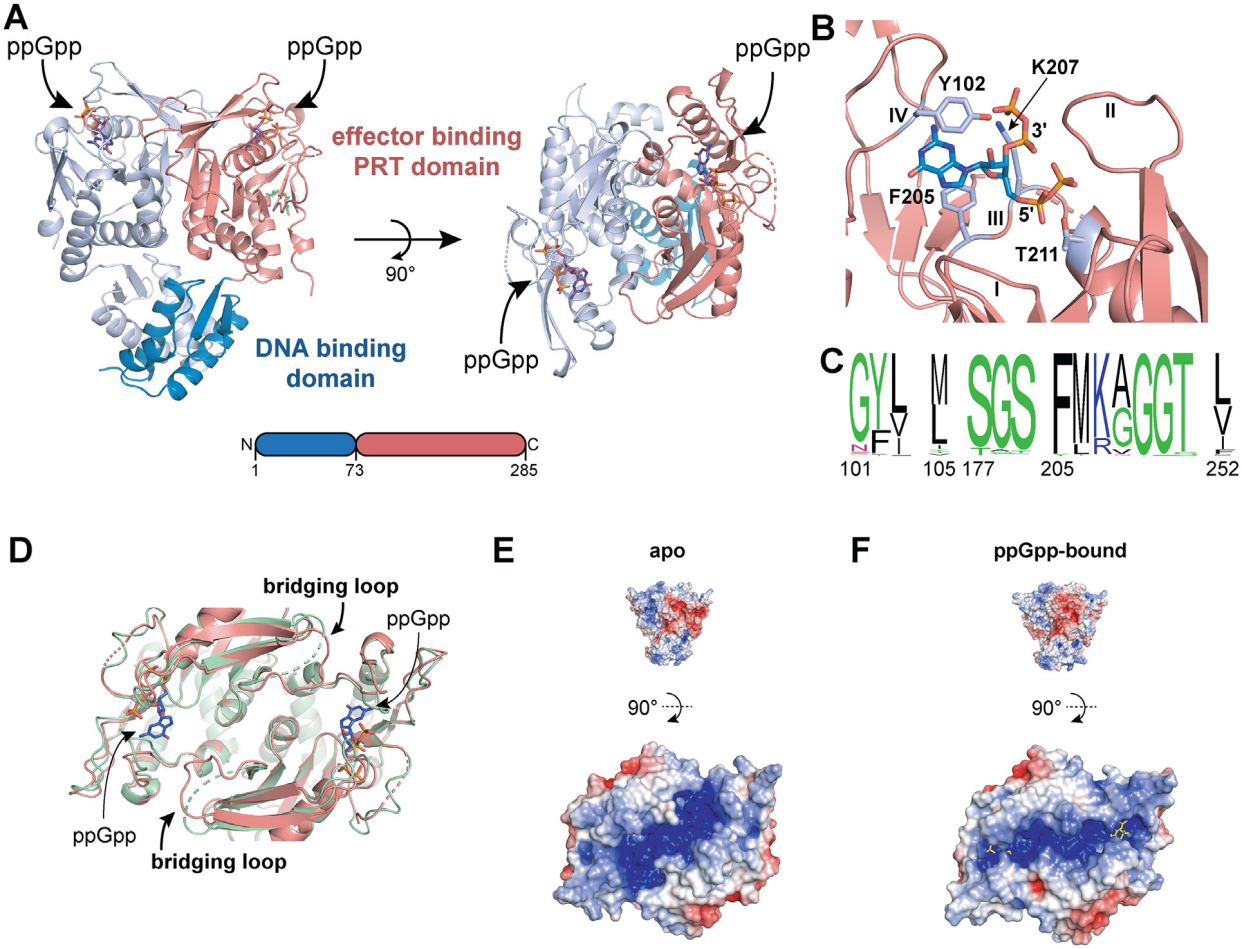
ppGpp binds to the effector binding domain of PurR. (**A**) *B. subtilis* PurR dimer crystallized with ppGpp. The N-terminal DNA binding domain is blue and the C-terminal effector binding phosphoribosyltransferase (PRT) domain is salmon. (Right) Structure from left rotated 90° to show the ppGpp binding pockets on the effector domains. (**B**) ppGpp binding pocket with select interacting residues indicated in silver. Loops I–IV of the PRT binding pocket are labeled. See Supplementary Figure S1B for an omit electron density map. (**C**) Frequency logo of the (p)ppGpp binding residues from 938 PurR proteins homologous to *B. subtilis* PurR. The logo was made with WebLogo (UC Berkeley; https://weblogo.berkeley.edu/logo.cgi). (**D**) Overlay of apo PurR (green; PDB ID 1O57) and ppGpp-bound PurR (salmon). Bridging loop that is unresolved in apo but not with ppGpp is indicated. (**E**, **F**) Poisson-Boltzmann continuum electrostatics of the effector binding domain of apo (E) and ppGpp-bound (F) PurR. The scale of electrostatic potential is –5 (red) to + 5 (blue).

We next solved a co-structure of ppGpp bound to *B. subtilis* PurR to 2.45 Å resolution (Figure 2A and Supplementary Table S2). *B. subtilis* PurR comprises two domains, an N-terminal winged helix-turn-helix domain for DNA binding and a C-terminal effector binding domain. In contrast to *E. coli* PurR whose effector binding domain binds purine bases and is structurally similar to a ribose binding protein, the Firmicute PurR effector binding domain is similar to the phosphoribosyltransferase (PRT) class of enzymes (Figure 2A) (21,53). In the structure of the *B. subtilis* PurR-ppGpp complex, ppGpp binds to the PRT domain in a pocket defined by three loops found in all PRT proteins (II, III, IV; loop numbering from PRT enzymes) (Figure 2A and B) (54). The guanine ring of ppGpp is engaged in π-stacking interactions, sandwiched between the aromatic side chains of residues Phe205 and Tyr102 (Figure 2B). A Y102A variant interacts with ^32^P-ppGpp more weakly than wild type, supporting a role for Tyr102 in ppGpp binding (Supplementary Figure S1A). The 5′-phosphates of ppGpp nestle into a pocket formed by loop III and an α-helix, where the backbone amides of loop III and the side chain of Thr211 coordinate the 5′-phosphates (Figure 2B). The 3′-phosphates extend upward and out of the binding pocket, where they interact with backbone amides of loop II and the side chains of Tyr102 and Lys207 (Figure 2B). The omit electron density for the 3′-phosphates is weaker than that of the 5′-phosphates (Supplementary Figure S1B) but can be unambiguously assigned to ppGpp when contoured down to lower sigma values. While (p)ppGpp is commonly co-crystallized with divalent cations (e.g. Mg^2+^ or Mn^2+^), we did not find evidence for metals in our structure. Accordingly, DRaCALA confirmed that Mg^2+^ is not required for nor interferes with the interaction between ^32^P-ppGpp and PurR (Supplementary Figure S1C).

The (p)ppGpp binding pocket among Firmicutes PurR homologs is highly conserved. We aligned the (p)ppGpp binding residues from 938 *B. subtilis* PurR homologs in the Pfam database (Figure 2C). Of the 15 residues involved in the interaction, 11 are nearly perfectly conserved, and variants in other sites are highly similar (Figure 2C). For example, residue 102 is a tyrosine in most PurR homologs, including *B. subtilis* PurR, while phenylalanine is at the homologous position in the others. Also, residue 207 is a lysine in most homologs, including *B. subtilis* PurR, and arginine is at the homologous position in the remainder. Loop III residues Phe205 and Gly209/Gly210/Thr211 are nearly universally conserved. ConSurf analysis across the whole protein for the 938 PurR homologs shows that the (p)ppGpp binding pocket is more highly conserved than the remainder of the protein, even more than the DNA binding domain (Supplementary Figure S1D).

Structural comparison between apo PurR and ppGpp-bound PurR does not reveal drastic conformational or electrostatic differences in the DNA binding domain (Supplementary Figure S2A-C). ppGpp binding does affect the effector binding domain, which is important for oligomeric interaction between PurR subunits. A loop that bridges the monomer-monomer interface of a PurR dimer is unresolved in apo PurR but is resolved in ppGpp-bound PurR, suggesting that ppGpp binding stabilizes the conformation of this loop (Figure 2D). The effect of this change is that ppGpp significantly alters the electrostatics of the effector binding domain by creating a positively charged channel different from the apo protein (Figure 2E and F). Since it has been hypothesized that DNA wraps around PurR to interact with the effector binding domain (55), it is possible that the altered electrostatic surfaces affect the interactions with the electronegative DNA backbone.

### (p)ppGpp competes with PRPP for binding PurR’s effector binding domain to alter PurR binding to promoter DNA

**Figure 3.**
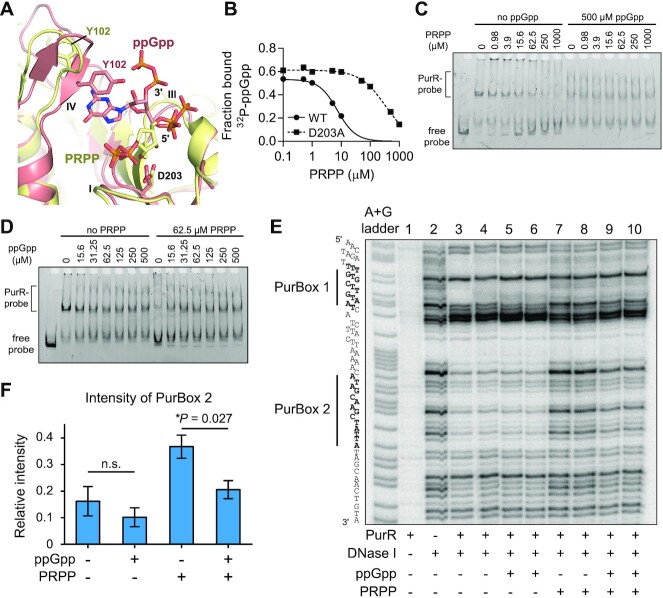
ppGpp competes with PRPP to allosterically regulate PurR-DNA interaction. (**A**) Overlay of PurR-ppGpp (salmon) with PurR-cPRPP (yellow; PDB ID 1P4A). The side chains of Y102 and D203 are shown. Loop II is hidden for clarity. (**B**) Competition between ^32^P-ppGpp (∼0.2 nM) and PRPP for binding to PurR and PurR D203A. Binding of ^32^P-ppGpp was measured with DRaCALA. Reactions were performed in technical triplicate, and error bars represent SEM. Error bars are not visible when their height is smaller than the height of the symbol. (**C**) EMSA showing PurR interaction with FAM-labeled 221 bp DNA with increasing PRPP concentrations and without ppGpp. (**D**) EMSA showing PurR interaction with DNA with increasing ppGpp concentration and with PRPP. The PurR concentration is 100 nM in (**C**) and (**D**). Similar EMSA results were observed with a non-labeled 202 bp probe from the same control region with a lower KCl concentration and no nonspecific DNA in the EMSA reaction (Supplementary Figure S3). (**E**) DNase I footprinting of PurR-PurBox interaction. ^32^P-labeled PurBox fragment was incubated with or without PurR (50 nM), ppGpp (1 mM), and PRPP (16 μM), and then briefly digested by DNase I (3.2 μg/ml). The digestion product was analyzed by electrophoresis. The uncropped gel is in Supplementary Figure S4. (**F**) Relative intensity of PurBox 2 in PurR-PurBox DNA footprint. Relative intensity is the intensity of the PurBox 2 area normalized to the intensity of a reference area (raw data in Supplementary Figures S5 and S6). Error bars represent standard error of the mean for 4 replicates. A two-tailed two-sample equal-variance Student's *t* test was performed between samples for statistical significance (*P ≤ 0.05; n.s. *P* > 0.05).

The effector binding domain of PurR is homologous to PRT enzymes, which catalyze phosphoribosyl transfer reactions with PRPP as a substrate. The PRT domain of PurR also binds PRPP but does not have enzymatic activity. PRPP binding instead de-represses the PurR regulon (20). The ppGpp binding site partially overlaps the PRPP binding site, as seen in an overlay of ppGpp and the PRPP analog, cPRPP, bound to PurR (Figure 3A) (55). Particularly, the 5′-phosphates of ppGpp overlap with the 5′-phosphate of PRPP in their interaction with loop III (Figure 3A). The overlap in this binding site suggests that they would compete for binding to PurR. Indeed, we found PRPP competes with ^32^P-ppGpp for binding PurR (Figure 3B). The binding pocket also contains interactions specific for each effector. PurR Tyr102 is flipped away from the pocket with PRPP bound and covers the guanine ring with ppGpp bound (Figure 3A). Asp203 sits below the ribose of PRPP but does not interact with ppGpp (Figure 3A). An alanine variant at this position reduces the ability of PRPP to compete with ppGpp by 100-fold (Figure 3B), but ^32^P-ppGpp binding is unaffected (Supplementary Figure S1A).

We next examined the role of (p)ppGpp/PRPP on PurR binding to a cognate binding sequence with electrophoretic mobility shift assays (EMSAs). We used two DNA probes amplified from the PurR-binding promoter of the *pur* operon in *B. subtilis*, which encodes the genes necessary for *de novo* IMP synthesis. One probe was a FAM-labeled 221 bp probe that we detected with FAM-specific fluorescence, and the other probe was an unlabeled 202 bp probe that we detected with a nonspecific SYBR Gold dye. We added PurR to the FAM-labeled probe at high salt concentrations in the presence of unlabeled nonspecific DNA competitors to ensure the specificity of the interaction (Figure 3C), or we added PurR to the unlabeled probe in the absence of nonspecific DNA at lower salt concentrations (Supplementary Figure S3). In both cases, we observed a complete probe shift and the formation of larger molecular weight complexes, indicative of the PurR repressor-promoter complex. The presence of increasing concentrations of PRPP results in loss of the larger molecular weight PurR-promoter complex and an increase in unbound DNA (Figure 3C, left). This agrees with previous observations that PRPP is an inducer (anti-repressor) of PurR (20). However, in the presence of ppGpp (500 μM), PRPP no longer effectively prevents PurR-DNA interaction, and only a small portion of unbound probe was observed even at the highest PRPP concentration (Figure 3C, right). We also performed EMSA by titrating an increasing amount of ppGpp (Figure 3D, left). ppGpp on its own does not increase PurR-promoter association. However, in the presence of PRPP (62.5 μM), ppGpp effectively antagonizes PRPP, increasing PurR-promoter association (Figure 3D, right). These results suggest that ppGpp maintains PurR-DNA interaction and prevents PRPP from de-repressing PurR regulation.

To clearly address the effect of (p)ppGpp on PurR-DNA binding, we performed a DNA footprinting assay using the *pur* promoter DNA probe (Figures 3E, S4, S5, and S6 and quantification in Figure 3F). This promoter DNA probe included the two 14-bp inverted repeats, termed PurBoxes (Figure 3E), that each bind a PurR dimer (56). The unbound probe is susceptible to DNase I digestion, including the PurBox regions (Figure 3E, lane 2). Addition of PurR (Figure 3E, lanes 3 and 4) led to significant protection of the PurBoxes against DNase I cleavage, indicating PurR binding to DNA. Addition of PRPP, even at low concentration (Figure 3E, lanes 7 and 8), led to an increase of PurBox intensities, particularly in the PurBox 2 region. This indicates deprotection of this region due to PRPP-mediated PurR de-repression. Addition of ppGpp itself had little effect on PurBox 2 intensity (Figure 3E, lanes 5 and 6), but addition of ppGpp in the presence of PRPP alleviated PRPP’s effect on increasing the PurBox 2 intensity (Figure 3E, lanes 9 and 10). These results indicate that ppGpp works as an anti-inducer against PRPP to maintain PurR-PurBox binding.

Overall, we conclude that (p)ppGpp binds PurR at an effector binding pocket distal to the DNA binding domain and competes with the inducer ligand PRPP to enhance the PurR-DNA interaction. Because PRPP is a key intermediate for nucleotide biosynthesis, it is always present *in vivo*. Based on our LC-MS analysis and metabolite quantification from the literature (31,32), we estimate PRPP to be ∼0.7 mM during amino acid starvation and ∼1.29 mM during nutrient replete growth (Supplementary Figure S7). These concentrations of PRPP are more than sufficient to saturate PurR *in vitro* (Figure 3C), suggesting that if PurR is relying on PRPP alone for its regulation, then its regulon should always be induced *in vivo*. This apparent contradiction between high concentrations of PRPP and its regulatory role has been characterized as an ‘enigma’ (47). (p)ppGpp may help to resolve this enigma for PurR by antagonizing PRPP, which enhances PurR DNA binding and allows PurR to function as an effective repressor during conditions that lead to (p)ppGpp induction, such as upon amino acid starvation.

### Defining the PurR binding sites in the *B. subtilis* genome

**Figure 4.**
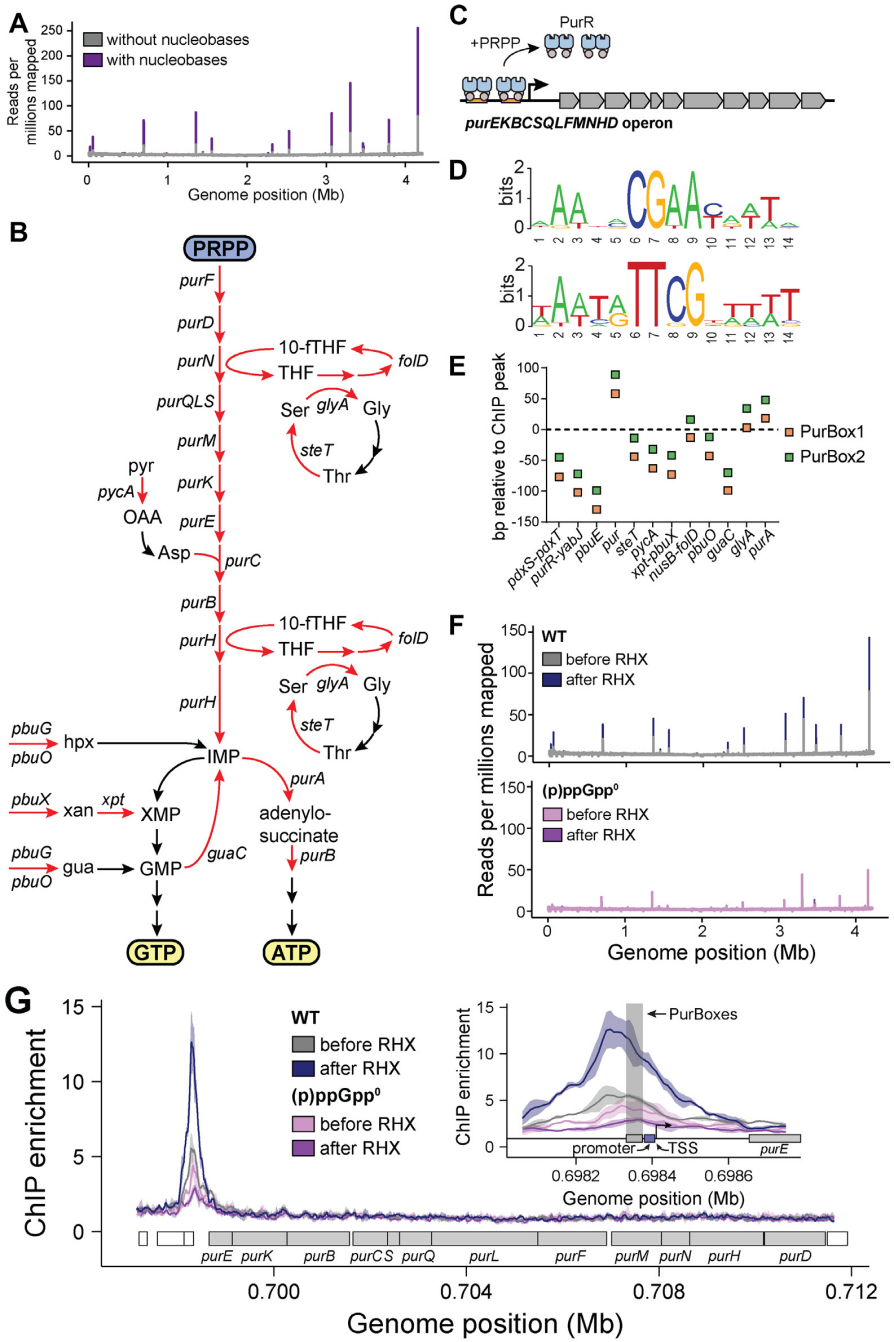
(p)ppGpp enhances PurR-DNA interaction during stress response. (**A**) Genome-wide view of mean PurR ChIP reads per million reads from *B. subtilis* grown with or without the nucleobases adenine, cytosine, guanine, and uracil. (**B**) Schematic of the GTP and ATP synthesis pathway in *B. subtilis*. Red arrows indicate PurR-controlled steps, as deduced from the respective genes being downstream of a PurR binding site. 10-fTHF = 10-formyl tetrahydrofolate; ser = serine; gly = glycine; thr = threonine; pyr = pyruvate; OAA = oxaloacetate; asp = aspartate; hpx = hypoxanthine; xan = xanthine; gua = guanine. Placements of *pycA* and *steT* in this pathway are inferred based on enzyme function and have not been verified. (**C**) PurR represses the 12-gene *pur* operon in *B. subtilis*, and PRPP is an inducing ligand. (**D**) Sequence logos of PurBox1 (top) and PurBox2 (bottom) sequences from 12 of the 15 PurR ChIP peaks. Logos were created using WebLogo (https://weblogo.berkeley.edu/logo.cgi). See Supplementary Figure S10 for complete sequences. (**E**) A plot showing the distance of PurBoxes from the ChIP enrichment peaks. The peak is represented by the dotted horizontal line at Y = 0. Peak location was determined at 10 bp resolution from the PurR ChIP sample obtained from (p)ppGpp-induced WT *B. subtilis*. The distance was calculated from the center of each PurBox (7th nt out of 15 nt PurBox) to the peak location. (**F**) Genome-wide view of mean PurR ChIP reads per million reads in WT and (p)ppGpp^0^ before and after arginine hydroxamate (RHX) treatment. Before RHX are the same data as without nucleobases in A. (**G**) PurR ChIP enrichment at the *pur* operon in WT and (p)ppGpp^0^. The solid trace is the mean enrichment of biological triplicate, and SD is shown as the shaded region. The inset shows the PurBox sites (shaded box), −10 and −35 promoter sequences (blue box), and the transcription start site (arrow) upstream of the *pur* operon. The sequence for this site is shown in Supplementary Figure S12.

(p)ppGpp binding to PurR and enhancing PurR–DNA interaction raised the possibility that *in vivo* (p)ppGpp accumulation during amino acid starvation directly regulates the PurR regulon. Since the PurR regulon in *B. subtilis* had not been systematically characterized, we turned to ChIP-seq to fully document PurR binding sites. We raised polyclonal antibodies against untagged *B. subtilis* PurR (Supplementary Figure S8) and collected ChIP samples from *B. subtilis* cells grown under a variety of conditions: with or without exogenous nucleobases and with or without amino acid starvation (Table 1). We first assessed ChIP-seq data with nucleobases to identify PurR binding sites genome-wide (Figure 4A). ChIP-seq identified 15 PurR binding sites (Figure 4A and 4B, Supplementary Figure S9, and Table 1), 9 of which had been previously identified (19). As expected, PurR enrichment was increased at all binding sites with nucleobases (Figure 4A). These PurR binding sites control expression of more than 30 genes, the majority of which are involved in purine nucleotide synthesis (Figure 4A). For example, PurR binds upstream of the 12-gene *pur* operon (*purEKBCSQLFMNHD*), which encodes all proteins necessary for *de novo* synthesis of the GTP/ATP precursor IMP from PRPP (Figure 4A and C). PurR also binds upstream of *pbuG* and *pbuO* (guanine/hypoxanthine permeases) and *xpt-pbuX* (xanthine phosphoribosyltransferase and xanthine permease), which altogether encode most proteins in the purine salvage pathway, and *purA* (the first dedicated step towards ATP synthesis) and *guaC* (GMP reductase that converts GMP to IMP). PurR controls expression of *nusB-folD* and *glyA*, which contribute to 10-formyltetrahydrofolate production necessary for *de novo* IMP synthesis (Figure 4B). Lastly, PurR binds upstream of its own promoter at the *purR-yabJ* operon.

**Table 1.** PurR binding sites in *B. subtilis* identified with ChIP-seq

		Wild type (PurR enrichment^a^)			(p)ppGpp^0^ (PurR enrichment)		
Gene/operon	Function	+ACGU^b^	−RHX^c^	+RHX	−RHX	+RHX	PurBox
***pdxS-pdxT***	pyridoxal 5-phosphate (PLP) synthetase	3.83 ± 0.72	1.63 ± 0.19	3.11 ± 0.52	1.36 ± 0.16	1.32 ± 0.10	Y
*purR-yabJ*	purine repressor PurR	11.30 ± 0.72	2.72 ± 0.43	7.19 ± 0.85	2.31 ± 0.43	1.79 ± 0.31	Y
*pbuG*	hypoxanthine/guanine permease	21.92 ± 1.54	4.93 ± 0.46	8.19 ± 1.44	4.10 ± 0.98	2.81 ± 0.37	Y
*purEKBCSQLFMNHD*	*de novo* IMP synthesis	19.28 ± 1.15	5.55 ± 1.03	12.51 ± 1.95	3.65 ± 0.59	2.35 ± 0.44	Y
***steT***	serine/threonine exchanger transporter	42.10 ± 7.78	10.19 ± 1.46	26.37 ± 8.74	12.52 ± 5.95	5.28 ± 0.21	Y
***pycA***	pyruvate carboxylase	20.49 ± 3.56	4.60 ± 0.76	14.24 ± 3.66	2.68 ± 1.10	1.99 ± 0.32	Y
***bdbB***	thiol-disulfide oxidoreductase	3.31 ± 1.17	1.59 ± 0.07	3.40 ± 1.48	1.57 ± 0.48	2.03 ± 0.45	N
*xpt-pbuX*	XPRT and xanthine permease	13.35 ± 1.66	4.13 ± 0.53	9.97 ± 0.86	3.25 ± 0.88	2.45 ± 0.42	Y
*nusB-folD*	transcription antiterminator NusB and methylene tetrahydrofolate dehydrogenase (FolD)	25.81 ± 4.31	5.73 ± 0.73	15.40 ± 2.56	3.66 ± 0.67	3.67 ± 0.55	Y
*pbuO*	hypoxanthine/guanine permease	34.14 ± 4.04	5.62 ± 0.60	14.18 ± 0.24	5.94 ± 2.06	2.74 ± 0.46	Y
*guaC*	GMP reductase	56.08 ± 4.55	10.85 ± 2.23	17.11 ± 3.38	16.46 ± 5.37	7.80 ± 1.40	Y
***opuBA-BB-BC-BD / yvaV* ^d^**	choline/arsenocholine ABC transporter	9.64 ± 2.00	4.59 ± 0.39	9.71 ± 0.25	2.89 ± 0.68	5.91 ± 1.01	N
***opuCA-CB-CC-CD / yvbF* ^d^**	ABC transporter (glycine for betaine/carnitine/choline/arsenocholine)	6.09 ± 1.85	3.38 ± 0.30	6.82 ± 0.87	2.30 ± 0.45	2.72 ± 0.33	N
*glyA*	serine hydroxymethyltransferase	25.55 ± 6.91	5.77 ± 0.84	9.12 ± 1.46	6.10 ± 1.45	4.06 ± 0.18	Y
*purA*	adenylosuccinate synthetase	50.75 ± 11.74	20.13 ± 4.09	34.25 ± 1.18	15.34 ± 4.20	7.90 ± 1.80	Y

^a^Enrichment of reads in immunoprecipitated sample relative to input sample (see Materials and Methods). Data are the mean and standard deviation of biological triplicate of the maximum points of each of the ChIP peaks.

^b^ACGU refers to the nucleobases adenine, cytosine, guanine, and uracil.

^c^RHX is arginine hydroxamate, which mimics amino acid starvation.

^d^The slash indicates a gene being transcribed in the opposite direction immediately upstream of the *opu* operons. It is not known which gene's expression, if not both, PurR is controlling.

Gene/operon names in bold indicate new PurR regulatory sites identified in this study

Our ChIP-seq peaks include six regions not previously known to be PurR binding sites in *B. subtilis*. The promoters at three of these sites, *pdxS-pdxT*, *steT*, and *pycA*, contain the PurBoxes that were identified at the nine previously known PurR binding sites (Supplementary Figure S10 and Table 1) (19). A sequence logo shows that the PurBoxes at all 12 sites are characterized by a central CGAA motif (or TTCG for the inverted repeat) surrounded by A–T rich sequences, and the 14-bp PurBoxes are separated by a 16- or 17-bp spacer (Figure 4D and Supplementary Figure S10). Two of these new PurR binding sites can be linked to purine nucleotide metabolism. First, the gene *steT* encodes a serine/threonine exchanger that we hypothesize may be involved in 10-formyltetrahydrofolate regeneration (Figure 4B). This binding site is consistent with *steT* previously being predicted to be regulated by PurR in a global network analysis (57). Second, the gene *pycA* encodes pyruvate carboxylase, which can be linked to aspartate synthesis, a necessary metabolite for *de novo* IMP synthesis (Figure 4B). Unexpected binding sites include upstream of the *pdxS-pdxT* operon, which encodes the enzyme complex required for *de novo* PLP (vitamin B6) synthesis (58). There are at least 60 PLP-dependent proteins in *B. subtilis*, a majority of them involved in amino acid biosynthesis (59). Seven transcription factors bind PLP, including GabR which regulates γ-aminobutyrate utilization (60,61).

We also identified ChIP peaks at the *opuB* and *opuC* operons, which encode transporters involved in osmotic protection in *B. subtilis* (62,63). The final PurR binding site with the weakest ChIP enrichment is intragenic upstream of *bdbB*, encoding a thiol-disulfide reductase. However, canonical PurBoxes were not found at the *opuC*, *opuB*, or *bdbB* sites. Further investigation will be required to determine whether these are bona fide PurR binding sites or false positive ChIP-seq results.

Interestingly, for all sites with PurBoxes, the peak of PurR ChIP occupancy was offset from the PurBoxes (Figure 4E), suggesting that PurR protects DNA distal from the PurBox binding sequence, possibly through extended PurR oligomerization. Since PurR copy numbers range from 1000 to 3000 per cell in *B. subtilis* (64,65), there are ample PurR copies to extend beyond the PurBoxes at each binding site.

### (p)ppGpp induction following amino acid starvation enhances promoter occupancy of PurR in *B. subtilis*

Next, we examined PurR ChIP signals 10 min after amino acid starvation, which was mimicked using the drug arginine hydroxamate (RHX). RHX induces (p)ppGpp to millimolar levels in wild-type cells to turn on the stringent response (11). After RHX treatment, PurR ChIP signal increased at each PurR binding site in wild-type cells by an average of 2.2-fold (Figure 4F, Supplementary Figure S11, and Table 1). This included a 2.3-fold increase at the *pur* operon (Figure 4G and Table 1; see Supplementary Figure S12 for the *pur* operon promoter sequence with the PurR binding sites). On the other hand, the PurR ChIP signal remained unchanged or decreased slightly in a (p)ppGpp-null ((p)ppGpp^0^) mutant (Figure 4G, Supplementary Figure S11, and Table 1), indicating that the increased enrichment in wild-type cells is dependent on (p)ppGpp.

To test how (p)ppGpp affects PurR occupancy independently of RHX treatment side-effects, we accounted for PurR occupancy changes due to RHX itself. We first calculated the effect of RHX treatment on PurR occupancy for both wild type and (p)ppGpp^0^ separately by dividing the PurR enrichment after RHX by the enrichment before RHX treatment. Using these datasets with the effect of RHX accounted for, we were then able to determine the effect of (p)ppGpp induction on PurR enrichment by taking the ratio of wild type over (p)ppGpp^0^. We found a (p)ppGpp-dependent increase in PurR occupancy at all 12 sites with canonical PurBoxes (Supplementary Figure S13). These PurR binding sites in Table 1 in total control expression of 28 genes, suggesting that these genes’ expression is likely also controlled by (p)ppGpp.

### (p)ppGpp binding to PurR down-regulates the expression of purine biosynthesis genes during nutrient downshift

**Figure 5.**
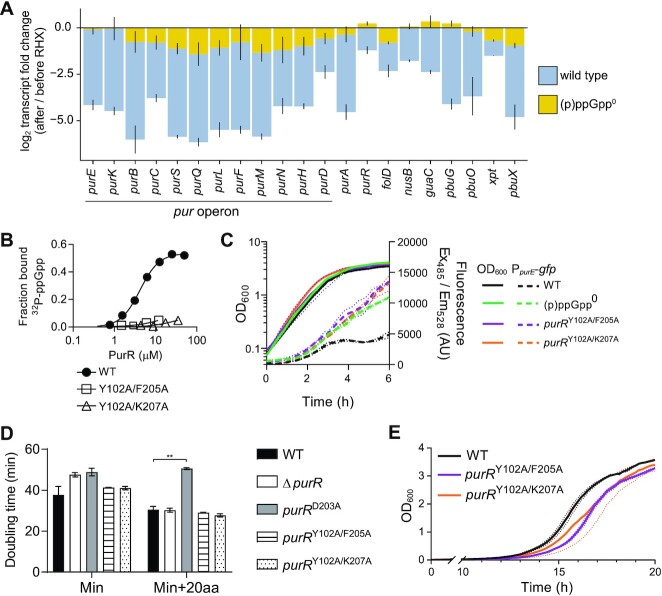
(p)ppGpp regulation of PurR is important for nutrient stress adaptation. (**A**) Change in transcript level of PurR-regulated genes after RHX treatment. In wild-type *B. subtilis*, genes are highly downregulated, with some genes in the *pur* operon being downregulated over 30-fold. In comparison, there is only a minor effect of RHX on expression of PurR-regulated genes in (p)ppGpp^0^. These data are from Kriel *et al.* (2014). Mean of triplicate ± SD is shown. (**B**) DRaCALA binding curves showing ^32^P-ppGpp interaction with PurR Y102A/F205A and Y102A/K207A. Experiments were performed in technical triplicate. Error bars representing SEM may be shorter than the height of the symbols. (**C**) Expression of a P*_purE_*-GFP reporter in wild-type, (p)ppGpp^0^, *purR*^Y102A/F205A^, and *purR*^Y102A/K207A^*B. subtilis*. (**D**) Doubling times of *B. subtilis* wild type, Δ*purR*, *purR*^D203A^, *purR*^Y102A/F205A^, and *purR*^Y102A/K207A^ in minimal medium (Min) and Min supplemented with 20 amino acids (Min + 20aa). An unpaired parametric two-tailed t-test with Welch's correction was used to compare wild type and mutants’ doubling times (** *P* ≤ 0.01; all other pairings are not significant, *P* > 0.05). (**E**) Growth of wild type, *purR*^Y102A/F205A^ and *purR*^Y102A/K207A^ in minimal medium following a nutrient downshift from Min + 20aa to Min media. All growth experiments were performed in biological triplicate, and error bars are SEM of triplicate. For OD and fluorescence curves, error bars are represented by dotted lines.

Next, we directly tested the hypothesis that (p)ppGpp increases repression of the PurR-regulated genes during nutrient adaptation in *Bacillus* cells. We analyzed our microarray-based transcriptomic profiles of *B. subtilis* cells under amino acid starvation (18). Strikingly, we observed that almost all the PurR regulated genes, including the *pur* operon, are downregulated up to 30-fold following amino acid starvation (Figure 5A and Supplementary Figure S14, Table 2). This downregulation is dependent on (p)ppGpp because little to no downregulation is observed in (p)ppGpp^0^ cells. These data suggest that (p)ppGpp accumulation not only enhances PurR binding, but strongly downregulates the expression of genes involved in purine nucleotide synthesis through both *de novo* and salvage pathways.

**Table 2.** Change in transcription from PurR-regulated genes after arginine hydroxamate treatment

Gene	Wild type (log_2_ fold change)	(p)ppGpp^0^ (log_2_ fold change)
*purE*	−4.15 ± 0.28	−0.1 ± 0.18
*purK*	−4.48 ± 0.22	−0.04 ± 0.62
*purB*	−6.01 ± 0.75	−0.75 ± 0.59
*purC*	−3.78 ± 0.22	−0.79 ± 0.38
*purS*	−5.86 ± 0.09	−1.12 ± 0.31
*purQ*	−6.15 ± 0.23	−1.41 ± 0.63
*purL*	−5.48 ± 0.4	−1.08 ± 0.42
*purF*	−5.49 ± 0.21	−0.78 ± 0.96
*purM*	−5.85 ± 0.17	−1.35 ± 0.47
*purN*	−4.21 ± 0.43	−1.2 ± 0.71
*purH*	−4.22 ± 0.15	−0.99 ± 0.5
*purD*	−2.38 ± 0.36	−0.58 ± 0.31
*purA*	−4.53 ± 0.42	−0.36 ± 0.42
*purR*	−1.2 ± 0.21	0.23 ± 0.11
*nusB*	−1.77 ± 0.07	0.07 ± 0.15
*folD*	−2.32 ± 0.34	−0.81 ± 0.08
*guaC*	−2.38 ± 0.11	0.33 ± 0.33
*pbuG*	−4.11 ± 0.29	0.23 ± 0.17
*pbuO*	−3.68 ± 1.03	−0.2 ± 0.28
*xpt*	−1.5 ± 0.01	−0.69 ± 0.06
*pbuX*	−4.8 ± 0.67	−0.96 ± 0.14
*steT*	−4.12 ± 0.59	−0.73 ± 0.29
*pycA*	1.65 ± 0.47	−0.7 ± 0.08
*pdxS*	−0.97 ± 0.35	−0.88 ± 0.08
*pdxT*	−0.87 ± 0.19	−0.87 ± 0.17

Log_2_ fold change in transcription from genes downstream of promoters with PurR-binding PurBoxes in *B. subtilis*. Note that *pdxS* and *pdxT* are downregulated in both wild type and (p)ppGpp^0^. This could be due to media differences between this study and Kriel *et al.* (2014) but requires further investigation. Data are mean ± SD. These data are adapted from Kriel *et al.* (2014).

To evaluate the role of (p)ppGpp on PurR regulation specifically, we engineered two PurR variants, PurR^Y102A/F205A^ and PurR^Y102A/K207A^. Based on our structural data, these two variants have alterations in residues that interact specifically with ppGpp but not PRPP. We verified that both variants no longer bind to (p)ppGpp (Figure 5B), yet still bind to DNA probes and their binding to DNA is still inhibited by PRPP (Supplementary Figure S15). We introduced the corresponding mutations into the *B. subtilis* genome at the endogenous *purR* locus to create (p)ppGpp-refractory, separation of function mutants of *purR*. Next, we engineered a GFP reporter controlled by the *pur* operon promoter and evaluated its expression. Using this reporter, we found that wild-type cells, but not the (p)ppGpp° cells, downregulate the expression of the *pur* reporter during entry into stationary phase (Figure 5C and Supplementary Figure S16A). The (p)ppGpp-refractory *purR* mutants both fail to downregulate the *pur* reporter, strongly supporting our hypothesis that (p)ppGpp interaction with PurR is necessary for repression of the PurR regulon during nutrient downshift.

Next, we examined the consequence of PurR-mediated transcriptional regulation of purine nucleotide synthesis on cell growth and nutrient transitions. We compared a strain with uninducible PurR repression (*purR*^D203A^, which lacks the ability to bind PRPP) (66,67), a Δ*purR* strain, and the (p)ppGpp-refractory *purR* mutant strains. All strains grew slowly in minimal medium (Figure 5D). However, in a richer medium supplemented with amino acids, all but the uninducible *purR*^D203A^ decreased their doubling times (Figure 5D, WT versus *purR*^D203A^*P* = 0.003). The doubling times of the Δ*purR* and (p)ppGpp-refractory *purR* mutants were not significantly different from that of wild type. This is expected because cells have only basal levels of (p)ppGpp in amino acid replete medium, thus the PurR regulon is expected to be induced by PRPP. The uninducible *purR* mutant displays comparative loss of fitness, suggesting that PurR regulon induction is important for growth under amino acid replete conditions.

Finally, we tested the importance of this interaction for cellular fitness on nutrient stress adaptation. We grew the (p)ppGpp-refractory *purR* mutant strains in a medium replete with amino acids and downshifted them into a medium lacking amino acids through a series of washes. We then followed outgrowth of the strains in minimal media. Both *purR*^Y102A/F205A^ and *purR*^Y102A/K207A^ had a lag time about one hour longer than wild type during outgrowth in minimal media following downshift (Figure 5E). We also performed a similar experiment where we downshifted the same strains from rich medium to minimal medium but resuspended them in rich medium again after the downshift. Even this transient (∼15–30 min) downshift was sufficient to increase the lag time of the (p)ppGpp-refractory *purR* mutant strains by about 30 min relative to wild type (Supplementary Figure S16B). A similar result was observed with Δ*purR*, which is not able to repress *de novo* purine synthesis during a downshift (Supplementary Figure S16C). Therefore, (p)ppGpp regulation of PurR promotes adaptation to changes in amino acid availability for *B. subtilis*.

## DISCUSSION

Transcription factors can relay environmental signals to transcriptional regulation through allosteric regulation by effector ligands. Here we discovered the first example of the ubiquitous alarmone (p)ppGpp acting as an effector for a transcription repressor. Our findings also demonstrate a direct regulation of transcription by (p)ppGpp in Firmicutes. (p)ppGpp binds the transcription repressor PurR, which controls purine nucleotide biosynthesis by binding to multiple promoter sites in *B. subtilis*. (p)ppGpp competes with the PurR inducer PRPP for a binding pocket in the PurR effector binding domain, thus allosterically increasing PurR binding to the promoter DNA. This allows (p)ppGpp to serve as an anti-inducer to strongly repress both *de novo* and salvage purine nucleotide synthesis. Our discovery thus reveals a new regulatory pathway through which purine biosynthesis gene expression is inhibited during amino acid starvation to enhance bacterial adaptation to fluctuating environmental conditions.

### (p)ppGpp as a classical effector of a transcription regulator

Our study provides the first example of the well-characterized alarmone (p)ppGpp acting as an effector of a transcription repressor. Transcription factors often bind effectors that relay external signals to internal responses, allowing a cell to modulate its physiology to adapt to environmental changes. Well-known examples include LacI binding allolactose to derepress the *lac* operon when lactose is available (68), and TrpR binding tryptophan to enhance repression of the *trp* operon when tryptophan is available (69,70). Many nucleotide second messengers also bind transcription factors to relay signals. cAMP binds CRP as a co-activator to signal low carbon availability (5). c-di-GMP binds FleQ in *P. aeruginosa* to regulate flagella expression and exopolysaccharide synthesis (71), VpsT in *Vibrio cholerae* to regulate biofilm formation (72), and BldD in *Streptomyces* to regulate development (7). c-di-AMP binds DarR in *M. tuberculosis* to influence ion and membrane homeostasis (8). All these examples include cyclic nucleotides. However, our findings broaden regulation of transcription factors to include a linear nucleotide, the ‘magic spot’ alarmone (p)ppGpp. It conforms to the fundamental principle of the genetic switch in which cellular signals allosterically regulate a transcription factor, i.e. a small molecule effector binds to a regulatory domain of the transcription factor to affect its binding to cognate operator/promoter DNA. Our study provides the first example of (p)ppGpp as such a classical effector.

Our discovery that (p)ppGpp binds PurR makes it the first case of (p)ppGpp directly regulating transcription in Firmicutes and only the third structurally-characterized example of (p)ppGpp regulation of transcription machines. In *E. coli*, (p)ppGpp binds the RNA polymerase at two sites, the interface of ω and β′ and the interface of β′ and the transcription factor DksA (14,41,42). By binding these sites, (p)ppGpp is proposed to influence RNA polymerase interdomain interaction and stabilize DksA-RNA polymerase interactions (14). In *Francisella tularensis*, (p)ppGpp binds to the interface of the heterodimeric MglA-SspA complex, which interacts with the RNA polymerase to recruit the transcriptional activator PigR (13). In Firmicutes, (p)ppGpp does not operate by either of these mechanisms. (p)ppGpp binds PurR to make the repressor inaccessible to the PRPP inducer, thus enhancing PurR binding to PurBox 2 (Figure 3E). The PurBoxes at the *pur* operon are just upstream of the -35 promoter element (Figure 4G and Supplementary Figure S12) (56). We have shown by ChIP-seq that PurR protects extensive promoter regions beyond PurBox 1 and PurBox 2, and the PurR ChIP signal overlaps with the −35 to −10 promoter elements (Figure 4G). Therefore, we propose that (p)ppGpp allows PurR to remain bound to the promoter despite the cellular presence of PRPP, thus enhancing its transcription repression of purine biosynthesis operons by blocking access of the RNA polymerase (Figure 5A). In all, (p)ppGpp regulates each of these transcription factors differently in different bacterial species, revealing that there is significant diversity in the effects of (p)ppGpp on transcriptional regulation.

There is no structural information on how apo PurR interacts with DNA, but substantial indirect evidence points to DNA wrapping around a PurR dimer and PurR nucleating on the DNA. At the *pur* operon, for example, two PurBoxes each bind a PurR dimer. However, six PurR dimers can associate with this region of DNA (55,56). At over 1000 PurR copies per cell (64,65), PurR far outnumbers its 15 binding sites. It is possible that additional PurR copies are nucleating on the DNA outside of the PurBoxes. Accordingly, the PurR DNase I footprint at the *pur* operon has been shown to extend beyond the PurBoxes themselves, with about 60 bp upstream and 20 bp downstream protected by PurR (20,46). This is also confirmed in our ChIP-seq data, where the PurBoxes are offset from the highest point of ChIP enrichment at nearly all the PurR binding sites (Figure 4E). Also, PurR binding to DNA induces positive supercoiling, suggesting the PurR causes broader topological changes to DNA (56).

Although our data support the function of (p)ppGpp as a competitor for the PurR effector PRPP, thus serving as an anti-inducer, or anti-anti-repressor in vivo, we cannot rule out a direct effect of (p)ppGpp on PurR−DNA interaction. Structural comparison suggests that (p)ppGpp binding to PurR affects its homo-oligomerization via a bridging loop (Figure 2D), which is critical for transcription factors’ function. Furthermore, PurR has positively charged surfaces at both the DNA binding domain and in the PRT domain near the effector binding pocket, supporting a proposal that the DNA can wrap around PurR (21). (p)ppGpp binding the PRT domain may enhance this wrapping of the DNA and nucleation of PurR on the DNA fragment. Further structural work is needed to characterize the interaction of PurR with DNA and the effects of (p)ppGpp and PRPP on this interaction.

It is now apparent that multiple bacterial phyla have evolved ways to use (p)ppGpp to directly regulate transcription. (p)ppGpp regulons have been recently delineated in Proteobacteria. (p)ppGpp's interaction with RNA polymerase allows it to regulate over 700 genes in *E. coli*, and its binding to MglA-SspA-PigR can upregulate at least 16 genes of the *Francisella* pathogenicity island (13,15,73,74). In Firmicutes, (p)ppGpp was previously only associated with indirect changes in gene expression, many of them likely due to (p)ppGpp's regulation of nucleotide enzymes and depletion of GTP (18). These include downregulation of ribosomal RNA which is controlled by concentration of the initiating nucleotide GTP (17) and genes in the *B. subtilis* CodY regulon which uses GTP as a co-repressor. (p)ppGpp indirectly activates the CodY regulon by decreasing GTP levels (75,76). Here, we have found that there is a (p)ppGpp-dependent increase in PurR binding upstream of 28 genes in *B. subtilis* after nutrient starvation (Supplementary Figure S13 and Table 1). Our work shows that as nutrient deprivation signals (p)ppGpp synthesis, (p)ppGpp binds to PurR to outcompete the inducer PRPP and represses the synthesis of purine nucleotides to conserve energy. Thus we now expand our understanding of (p)ppGpp transcriptional regulon in Firmicutes beyond indirect regulation.

### A conserved (p)ppGpp-binding motif shared among enzymes and a transcription factor

**Figure 6.**
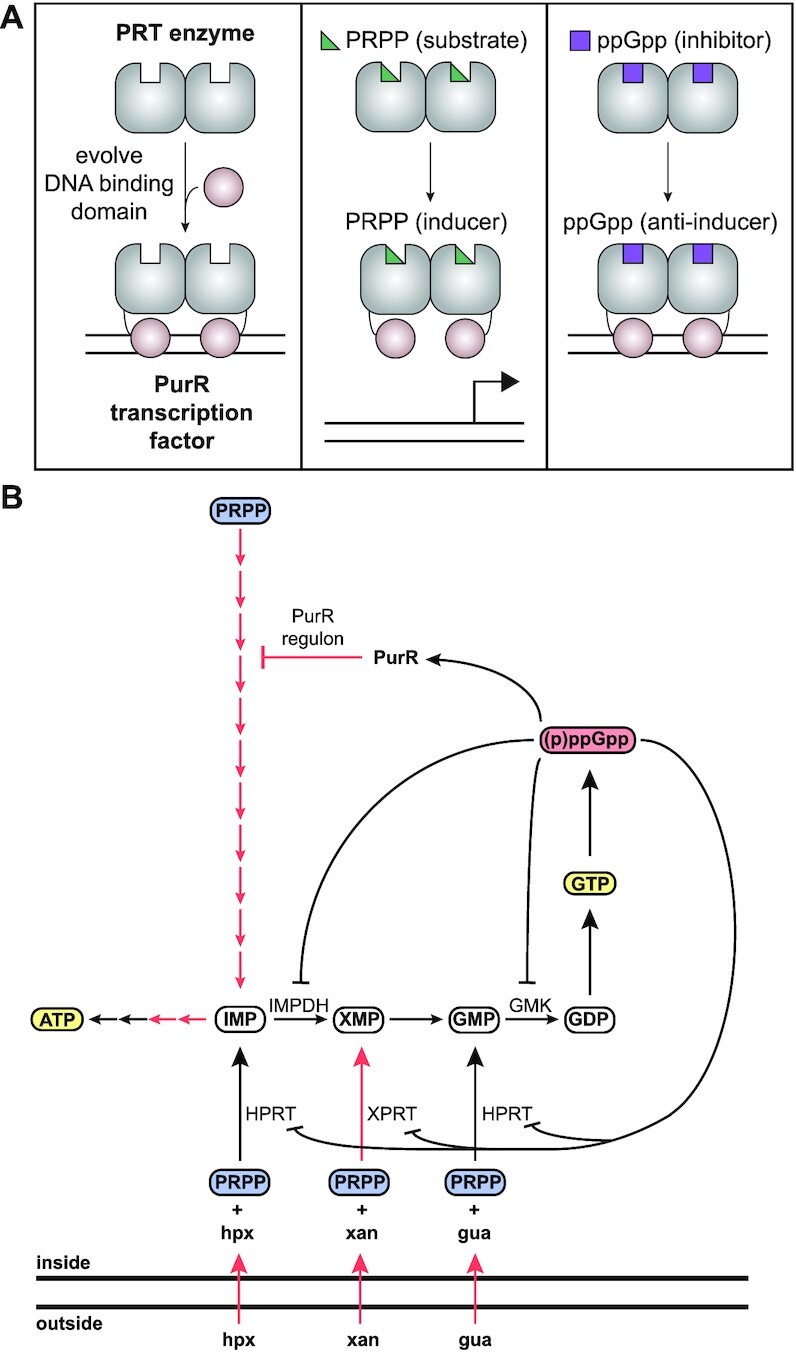
Models of (p)ppGpp regulation of PurR and global (p)ppGpp regulation of purine nucleotide synthesis in *B. subtilis*. (**A**) Schematic of PurR evolution from PRT enzymes like XPRT. PurR repurposes the active site of PRT enzymes to serve as an effector binding pocket, which PRPP and ppGpp bind to allosterically regulate DNA binding. (**B**) GTP and ATP synthesis in *B. subtilis*. (p)ppGpp regulates GTP synthesis at multiple points, including inhibition of GTP synthesis enzymes (IMPDH, GMK, HPRT, XPRT) and binding the transcription factor PurR. Red arrows represent steps in the PurR regulon.

The nucleotide (p)ppGpp serves a highly pleiotropic role in protecting bacteria by interacting with a multitude of protein and riboswitch targets to remodel bacterial metabolomes, transcriptomes, and proteomes and to tune bacterial physiology for growth in varied environments (44,77–79). (p)ppGpp regulates its targets with a few relatively conserved direct binding motifs. In one case, (p)ppGpp binds to many GTPases through their GTP-binding motif (80–83). In another case, (p)ppGpp binds to a conserved PRPP-binding motif in the PRT fold in two enzymatic targets, the purine salvage enzymes HPRT and XPRT (26,27). The (p)ppGpp binding site on PurR is the third PRT site also found to bind (p)ppGpp (Figure 6A). Here, the PRT enzyme is repurposed to become an effector binding regulatory domain of a transcription factor, the substrate PRPP is repurposed to become an inducer, and the enzyme inhibitor (p)ppGpp is repurposed to become an anti-inducer (Figure 6A). This striking evolutionary strategy highlights how nature utilizes limited motifs to evolve diverse regulatory targets of (p)ppGpp, allowing it to serve as a master regulator of nutrient stress.

Despite PurR, HPRT and XPRT sharing the same (p)ppGpp binding motif, how ligand binding affects the function of each protein has diversified through distinct allosteric and oligomeric properties surrounding this binding pocket. For HPRT, robust (p)ppGpp inhibition is dependent on HPRT being a tetramer. Tetramerization allosterically opens the (p)ppGpp binding pocket by holding loop II at the dimer−dimer interface. Alteration of the dimer−dimer interface then allows diversification of (p)ppGpp regulation of HPRT across phylogenetically diverse organisms without changing the binding pocket (26). In XPRT, two (p)ppGpp binding sites face one another across an XPRT monomer-monomer interface in an XPRT dimer. (p)ppGpp binding creates an electrostatic network with the bridging loop of the other monomer, inducing XPRT dimerization, this enables a cooperativity of regulation with Hill coefficient of 2 (27). In this study, we have discovered a third allosteric property associated with this same motif. By binding its effector site on PurR, (p)ppGpp competes with an inducer of a transcription factor to enhance repression during stress.

Our work has also highlighted a broader (p)ppGpp binding motif shared among GTPases, PRTs, and other targets such as guanylate kinase (80,82,84,85). GTPases and guanylate kinase bind (p)ppGpp with a phosphate-binding loop (P-loop) motif, which is used to interact with nucleotides (86). While the PRT proteins lack the P-loop amino acid sequence, they are structurally reminiscent of the P-loop with a loop leading into the positive dipole of an α-helix. The 5′-phosphates of (p)ppGpp and PRPP fit into the pocket created by the loop and are coordinated by the loop's backbone (Figures 2D and 3A). This structural motif associated with (p)ppGpp binding may become more prevalent as more (p)ppGpp binding targets are characterized.

### Implication of (p)ppGpp-PRPP competition on nutritional regulation of PurR regulon

During the evolutionary co-opting of the PRT enzyme as the PurR effector binding regulatory domain, not only has (p)ppGpp binding been conserved, so has the (p)ppGpp-PRPP competition (Figure 6A). PRPP is the common substrate for HPRT and XPRT, and (p)ppGpp inhibits HPRT and XPRT by competing with PRPP (26,27). Here we have shown that (p)ppGpp also competes with PRPP to bind PurR. In all cases, PRPP is a signal to the cell for increased nucleotide synthesis: it is directly converted to nucleotides through purine salvage or it binds PurR to de-repress the *de novo* IMP synthesis pathway (47). (p)ppGpp competes with PRPP by binding the same binding site in each of these proteins, shutting down nucleotide synthesis.

The competition between (p)ppGpp and PRPP for PurR binding likely tunes transcription from the PurR regulon during nutrient replete and nutrient starved growth. During unstressed growth, PRPP concentrations range from hundreds of micromolar to millimolar and (p)ppGpp is at concentrations <50 μM (Supplementary Figure S7) (11,47). Since both PRPP and (p)ppGpp bind PurR with *K*_d_ ∼ 5 μM (Figure 1H) (47), in nutrient replete conditions PRPP levels dictate PurR binding and induce PurR-regulated genes for rapid synthesis of GTP and ATP. PRPP-mediated induction can be overridden by salvageable nucleobases, which repress the *de novo* purine operon by consuming PRPP upon conversion to nucleotides and by binding riboswitches that control select nucleotide synthesis genes (67,87).

During amino acid starvation, (p)ppGpp levels are elevated up to >1 mM, allowing (p)ppGpp to compete with PRPP and repress purine nucleotide synthesis. This not only conserves energy and resources used for transcription and translation of the PurR regulon, but enables the coordinated use of PRPP, which is a common precursor of nucleotides (GTP, ATP, UTP) and several amino acids (47). In *E. coli*, a mutant for which (p)ppGpp fails to inhibit purine nucleotide synthesis results in PRPP depletion due to over-production of purine nucleotides (88). (p)ppGpp inhibition of purine synthesis redirects PRPP from purine nucleotide synthesis to UTP and amino acid synthesis during starvation (88,89). We propose that, in Firmicutes, the direct competition between (p)ppGpp and PRPP for PurR, HPRT and XPRT (see Figure 6B) would serve the same purpose, allowing (p)ppGpp to coordinate the consumption of PRPP between purine synthesis and amino acid synthesis during starvation. Interestingly, the (p)ppGpp-PRPP competition in *B. subtilis* would allow maintenance of PRPP levels: when (p)ppGpp accumulates after amino acid starvation, it directly prevents PRPP depletion by inhibiting its consumption in purine salvage and by preventing PRPP from activating *de novo* IMP synthesis.

### (p)ppGpp controls *de novo* purine synthesis by targeting different protein factors between bacterial species

Our results reveal that (p)ppGpp regulates *de novo* purine synthesis across bacteria. The 11 reactions that synthesize IMP from PRPP are necessary for *de novo* synthesis of ATP and GTP. Until recently, there was no evidence that (p)ppGpp directly regulated this pathway in bacteria. In *E. coli*, it was recently shown that (p)ppGpp inhibits the activity of PurF, the first step in the pathway, and (p)ppGpp binding the RNA polymerase decreases the transcription of all the genes in the pathway (15,78). We have shown that (p)ppGpp regulation of *de novo* purine synthesis extends to Firmicutes, albeit by an entirely different mechanism. While Proteobacteria and Firmicutes have evolved different mechanisms for this regulation, they share the common theme of decreasing *de novo* synthesis upon (p)ppGpp induction. This theme is likely conserved across other bacteria as well.

With our data, we can begin building a model of (p)ppGpp regulating purine nucleotide synthesis at multiple checkpoints in Firmicutes. As we have shown, it regulates *de novo* and salvage nucleotide synthesis genes via its interaction with PurR (Figure 6B). (p)ppGpp also inhibits the activities of nucleobase salvage enzymes HPRT and XPRT (11,26,27), and it potently inhibits guanylate kinase activity, which catalyzes GMP to GDP (Figure 6B) (85). Altogether, purine nucleotide synthesis has evolved to be sensitive to (p)ppGpp regulation at a majority of the synthesis steps. Since purine nucleotides are often a signal to increase growth, (p)ppGpp counteracts this during stressful conditions to control growth and enable survival.

## DATA AVAILABILITY

ChIP-seq analysis pipeline and scripts are available at https://github.com/bwanderson3/2021_PurR_ChIP_analysis.git.

Atomic coordinates and structure factors for the reported crystal structure have been deposited with the Protein Data Bank under accession number 7RMW. ChIP-seq datasets have been deposited with the NCBI Gene Expression Omnibus under GSE185164.

## Supplementary Material

gkab1281_Supplemental_Files

## References

[1] Fahmi T. , Port G.C., Cho K.H. C-di-AMP: an essential molecule in the signaling pathways that regulate the viability and virulence of gram-positive bacteria. Genes (Basel). 2017; 8:197. 10.3390/genes8080197 PMC5575661 28783096

[2] Gallagher K.A. , Schumacher M.A., Bush M.J., Bibb M.J., Chandra G., Holmes N.A., Zeng W., Henderson M., Zhang H., Findlay K.C. et al. c-di-GMP arms an Anti-σ to control progression of multicellular differentiation in streptomyces. Mol. Cell. 2020; 77:586–599. 31810759 10.1016/j.molcel.2019.11.006 PMC7005675

[3] Hengge R. Principles of c-di-GMP signalling in bacteria. Nat. Rev. Microbiol. 2009; 7:263–273. 19287449 10.1038/nrmicro2109

[4] Kalia D. , Merey G., Nakayama S., Zheng Y., Zhou J., Luo Y., Guo M., Roembke B.T., Sintim H.O. Nucleotide, c-di-GMP, c-di-AMP, cGMP, cAMP,(p)ppGpp signaling in bacteria and implications in pathogenesis. Chem. Soc. Rev. 2013; 42:305–341. 23023210 10.1039/c2cs35206k

[5] Sassone-Corsi P. Transcription factors responsive to cAMP. Annu. Rev. Cell Dev. Biol. 1995; 11:355–377. 8689562 10.1146/annurev.cb.11.110195.002035

[6] Tao F. , He Y.W., Wu D.H., Swarup S., Zhang L.H. The cyclic nucleotide monophosphate domain of xanthomonas campestris global regulator clp defines a new class of cyclic di-GMP effectors. J. Bacteriol. 2010; 192:1020–1029. 20008070 10.1128/JB.01253-09 PMC2812978

[7] Tschowri N. , Schumacher M.A., Schlimpert S., Chinnam N., Findlay K.C., Brennan R.G., Buttner M.J. Tetrameric c-di-GMP mediates effective transcription factor dimerization to control *streptomyces* development. Cell. 2014; 158:1136–1147. 25171413 10.1016/j.cell.2014.07.022 PMC4151990

[8] Zhang L. , Li W.H., He Z.G. DarR, a tetr-like transcriptional factor, is a cyclic di-AMP-responsive repressor in *mycobacterium**smegmatis*. J. Biol. Chem. 2013; 288:3085–3096. 23250743 10.1074/jbc.M112.428110 PMC3561532

[9] Ullman A. , Tillier F., Monod J. Catabolite modulator factor: a possible mediator of catabolite repression in bacteria. Proc. Natl. Acad. Sci. U.S.A. 1976; 73:3476–3479. 185615 10.1073/pnas.73.10.3476 PMC431138

[10] Cashel M. , Gentry D.R., Hernandez V.J., Vinella D The stringent response. *Escherichia coli* and *Salmonella typhimurium*: Cellular and Molecular Biology. 1996; 1458–1496.

[11] Kriel A. , Bittner A.N., Kim S.H., Liu K., Tehranchi A.K., Zou W.Y., Rendon S., Chen R., Tu B.P., Wang J.D. Direct regulation of GTP homeostasis by (p)ppGpp: a critical component of viability and stress resistance. Mol. Cell. 2012; 48:231–241. 22981860 10.1016/j.molcel.2012.08.009 PMC3483369

[12] Potrykus K. , Cashel M. (p)ppGpp: still magical?. Annu. Rev. Microbiol. 2008; 62:35–51. 18454629 10.1146/annurev.micro.62.081307.162903

[13] Cuthbert B.J. , Ross W., Rohlfing A.E., Dove S.L., Gourse R.L., Brennan R.G., Schumacher M.A. Dissection of the molecular circuitry controlling virulence in *francisella**tularensis*. Genes Dev. 2017; 31:1549–1560. 28864445 10.1101/gad.303701.117 PMC5630020

[14] Ross W. , Sanchez-Vazquez P., Chen A.Y., Lee J.H., Burgos H.L., Gourse R.L. ppGpp binding to a site at the RNAP-DksA interface accounts for its dramatic effects on transcription initiation during the stringent response. Mol. Cell. 2016; 62:811–823. 27237053 10.1016/j.molcel.2016.04.029 PMC4912440

[15] Sanchez-Vazquez P. , Dewey C.N., Kitten N., Ross W., Gourse R.L. Genome-wide effects on *Escherichia**coli* transcription from ppGpp binding to its two sites on RNA polymerase. Proc. Natl. Acad. Sci. U.S.A. 2019; 116:8310–8319. 30971496 10.1073/pnas.1819682116 PMC6486775

[16] Handke L.D. , Shivers R.P., Sonenshein A.L. Interaction of *Bac**illus**subtilis* CodY with GTP. J. Bacteriol. 2008; 190:798–806. 17993518 10.1128/JB.01115-07 PMC2223590

[17] Krásný L. , Gourse R. An alternative strategy for bacterial ribosome synthesis: *Bacillus**subtilis* rRNA transcription regulation. EMBO J. 2004; 23:4473–4483. 15496987 10.1038/sj.emboj.7600423 PMC526457

[18] Kriel A. , Brinsmade S.R., Tse J.L., Tehranchi A.K., Bittner A.N., Sonenshein A.L., Wang J.D. GTP dysregulation in *bacillus**subtilis* cells lacking (p)ppGpp results in phenotypic amino acid auxotrophy and failure to adapt to nutrient downshift and regulate biosynthesis genes. J. Bacteriol. 2014; 196:189–201. 24163341 10.1128/JB.00918-13 PMC3911124

[19] Saxild H.H. , Brunstedt K., Nielsen K.I. Definition of the *Bacillus subtilis* PurR operator using genetic and bioinformatic tools and expansion of the PurR regulon with *glyA*, *guaC*, *pbuG*, *xpt-pbuX*, *yqhZ-folD*, and *pbuO*. J. Bacteriol. 2001; 183:6175–6183. 11591660 10.1128/JB.183.21.6175-6183.2001 PMC100094

[20] Weng M. , Nagy P.L., Zalkin H. Identification of the *bacillus**subtilis pur* operon repressor. Proc. Natl. Acad. Sci. U.S.A. 1995; 92:7455–7459. 7638212 10.1073/pnas.92.16.7455 PMC41358

[21] Sinha S.C. , Krahn J., Shin B.S., Tomchick D.R., Zalkin H., Smith J.L. The purine repressor of *Bacillus**subtilis*: a novel combination of domains adapted for transcription regulation. J. Bacteriol. 2003; 185:4087–4098. 12837783 10.1128/JB.185.14.4087-4098.2003 PMC164869

[22] Kirsch R.D. , Joly E. An improved PCR-mutagenesis strategy for two-site mutagenesis or sequence swapping between related genes. Nucleic Acids Res. 1998; 26:1848–1850. 9512562 10.1093/nar/26.7.1848 PMC147442

[23] Vasantha N. , Freese E. Enzyme changes during *Bacillus**subtilis* sporulation caused by deprivation of guanine nucleotides. J. Bacteriol. 1980; 144:1119–1125. 6777366 10.1128/jb.144.3.1119-1125.1980 PMC294778

[24] Spizizen J. Transformation of biochemically deficient strains of *Bacillus**subtilis* by deoxyribonucleate. Proc. Natl. Acad. Sci. U.S.A. 1958; 44:1072–1078. 16590310 10.1073/pnas.44.10.1072 PMC528696

[25] Roelofs K.G. , Wang J., Sintim H.O., Lee V.T. Differential radial capillary action of ligand assay for high-throughput detection of protein-metabolite interactions. Proc. Natl. Acad. Sci. U.S.A. 2011; 108:15528–15533. 21876132 10.1073/pnas.1018949108 PMC3174574

[26] Anderson B.W. , Liu K., Wolak C., Dubiel K., She F., Satyshur K.A., Keck J.L., Wang J.D. Evolution of (p)ppGpp-HPRT regulation through diversification of an allosteric oligomeric interaction. Elife. 2019; 8:e47534. 31552824 10.7554/eLife.47534 PMC6783271

[27] Anderson B.W. , Hao A., Satyshur K.A., Keck J.L., Wang J.D. Molecular mechanism of regulation of the purine salvage enzyme XPRT by the alarmones pppGpp, ppGpp, and pGpp. J. Mol. Biol. 2020; 432:4108–4126. 32446804 10.1016/j.jmb.2020.05.013 PMC7323586

[28] Leslie A.G.W. The integration of macromolecular diffraction data. Acta Crystallogr. Sect. D Biol. Crystallogr. 2006; 62:48–57. 16369093 10.1107/S0907444905039107

[29] Adams P.D. , Afonine P.V., Bunkoczi G., Chen V.B., Davis I.W., Echols N., Headd J.J., Hung L.-W., Kapral G.J., Grosse-Kunstleve R.W. et al. PHENIX: a comprehensive Python-based system for macromolecular structure solution. Acta Crystallogr. Sect. D Biol. Crystallogr. 2010; 66:213–221. 20124702 10.1107/S0907444909052925 PMC2815670

[30] Yang J. , Anderson B.W., Turdiev A., Turdiev H., Stevenson D.M., Amador-Noguez D., Lee V.T., Wang J.D. The nucleotide pGpp acts as a third alarmone in Bacillus, with functions distinct from those of (p)ppGpp. Nat. Commun. 2020; 11:538. 33097692 10.1038/s41467-020-19166-1 PMC7584652

[31] Shi S. , Chen T., Zhang Z., Chen X., Zhao X. Transcriptome analysis guided metabolic engineering of bacillus subtilis for riboflavin production. Metab. Eng. 2009; 11:243–252. 19446032 10.1016/j.ymben.2009.05.002

[32] Bratbak G. , Dundas I. Bacterial dry matter content and biomass estimations. Appl. Environ. Microbiol. 1984; 48:755–757. 6508285 10.1128/aem.48.4.755-757.1984 PMC241608

[33] Ross W. , Gourse R.L. Analysis of RNA polymerase-promoter complex formation. Methods. 2009; 47:13–24. 18952176 10.1016/j.ymeth.2008.10.018 PMC2633133

[34] Bonocora R.P. , Wade J.T. Artsimovitch I. , Santangelo T.J. ChIP-Seq for genome-scale analysis of bacterial DNA- Binding Proteins. Bacterial Transcriptional Control: Methods and Protocols. 2015; 1276:NY Springer Science+Business Media, Inc 327–340. 10.1007/978-1-4939-2392-2_20 25665574

[35] Zhang Y. , Mooney R., Grass J., Sivaramakrishnan P., Herman C., Landick R., Wang J. DksA guards elongating RNA polymerase against ribosome-stalling-induced arrest. Mol. Cell. 2014; 53:766–778. 24606919 10.1016/j.molcel.2014.02.005 PMC4023959

[36] Kumar S. , Stecher G., Li M., Knyaz C., Tamura K. MEGA X: molecular evolutionary genetics analysis across computing platforms. Mol. Biol. Evol. 2018; 35:1547–1549. 29722887 10.1093/molbev/msy096 PMC5967553

[37] Nei M. , Kumar S. Molecular Evolution and Phylogenetics. 2000; Oxford Oxford University Press.

[38] Yu G. , Smith D.K., Zhu H., Guan Y., Lam T.T.Y. GGTREE: an r package for visualization and annotation of phylogenetic trees with their covariates and other associated data. Methods Ecol. Evol. 2017; 8:28–36.

[39] Ashkenazy H. , Abadi S., Martz E., Chay O., Mayrose I., Pupko T., Ben-Tal N. ConSurf 2016: an improved methodology to estimate and visualize evolutionary conservation in macromolecules. Nucleic Acids Res. 2016; 44:W344–W350. 27166375 10.1093/nar/gkw408 PMC4987940

[40] Landau M. , Mayrose I., Rosenberg Y., Glaser F., Martz E., Pupko T., Ben-Tal N. ConSurf 2005: the projection of evolutionary conservation scores of residues on protein structures. Nucleic Acids Res. 2005; 33:299–302. 10.1093/nar/gki370 PMC1160131 15980475

[41] Molodtsov V. , Sineva E., Zhang L., Huang X., Cashel M., Ades S.E., Murakami K.S. Allosteric effector ppGpp potentiates the inhibition of transcript initiation by dksA. Mol. Cell. 2018; 69:828–839. 29478808 10.1016/j.molcel.2018.01.035 PMC5837818

[42] Ross W. , Vrentas C.E., Sanchez-Vazquez P., Gaal T., Gourse R.L. The magic spot: a ppGpp binding site on *E**. coli* RNA polymerase responsible for regulation of transcription initiation. Mol. Cell. 2013; 50:420–429. 23623682 10.1016/j.molcel.2013.03.021 PMC3654024

[43] Liu K. , Bittner A.N., Wang J.D. Diversity in (p)ppGpp metabolism and effectors. Curr. Opin. Microbiol. 2015; 24:72–79. 25636134 10.1016/j.mib.2015.01.012 PMC4380541

[44] Corrigan R.M. , Bellows L.E., Wood A., Gründling A. ppGpp negatively impacts ribosome assembly affecting growth and antimicrobial tolerance in Gram-positive bacteria. Proc. Natl. Acad. Sci. U.S.A. 2016; 113:E1710–E1719. 26951678 10.1073/pnas.1522179113 PMC4812758

[45] Wang J.D. , Sanders G.M., Grossman A.D. Nutritional control of elongation of DNA replication by (p)ppGpp. Cell. 2007; 128:865–875. 17350574 10.1016/j.cell.2006.12.043 PMC1850998

[46] Ebbole D.J. , Zalkin H. Interaction of a putative repressor protein with an extended control region of the *Bacillus**subtilis pur* operon. J. Biol. Chem. 1989; 264:3553–3561. 2536750

[47] Hove-Jensen B. , Andersen K.R., Kilstrup M., Martinussen J., Switzer R.L., Willemoes M. Phosphoribosyl diphosphate (PRPP): biosynthesis, enzymology, utilization, and metabolic significance. Microbiol. Mol. Biol. Rev. 2017; 81:e00040-16. 28031352 10.1128/MMBR.00040-16 PMC5312242

[48] Sause W.E. , Balasubramanian D., Irnov I., Copin R., Sullivan M.J., Sommerfield A., Chan R., Dhabaria A., Askenazi M., Ueberheide B. et al. The purine biosynthesis regulator PurR moonlights as a virulence regulator in staphylococcus aureus. Proc. Natl. Acad. Sci. U.S.A. 2019; 116:13563–13572. 31217288 10.1073/pnas.1904280116 PMC6613142

[49] Rappu P. , Shin B.S., Zalkin H., Shin B.S.I.K. A role for a highly conserved protein of unknown function in regulation of *Bacillus subtilis* purA by the purine repressor. J. Bacteriol. 1999; 181:3810–3815. 10368157 10.1128/jb.181.12.3810-3815.1999 PMC93860

[50] Saxild H.H. , Nygaard P. Regulation of levels of purine biosynthetic enzymes in *Bacillus**subtilis*: effects of changing purine nucleotide pools. J. Gen. Microbiol. 1991; 137:2387–2394. 1722815 10.1099/00221287-137-10-2387

[51] Choi K.Y. , Zalkin H. Structural characterization and corepressor binding of the escherichia coli purine repressor. J. Bacteriol. 1992; 174:6207–6214. 1400170 10.1128/jb.174.19.6207-6214.1992 PMC207689

[52] Meng L.M. , Nygaard P. Identification of hypoxanthine and guanine as the co-repressors for the purine regulon genes of *Escherichia**coli*. Mol. Microbiol. 1990; 4:2187–2192. 2089227 10.1111/j.1365-2958.1990.tb00580.x

[53] Schumacher M.a , Choi K.Y., Zalkin H., Brennan R.G. Crystal structure of LacI member, PurR, bound to DNA: minor groove binding by alpha helices. Science. 1994; 266:763–770. 7973627 10.1126/science.7973627

[54] Sinha S.C. , Smith J.L. The PRT protein family. Curr. Opin. Struct. Biol. 2001; 11:733–739. 11751055 10.1016/s0959-440x(01)00274-3

[55] Bera A.K. , Zhu J., Zalkin H., Smith J.L. Functional dissection of the *Bacillus**subtilis pur* operator site. J. Bacteriol. 2003; 185:4099–4109. 12837784 10.1128/JB.185.14.4099-4109.2003 PMC164870

[56] Shin B.S. , Stein A., Zalkin H. Interaction of *Bacillus**subtilis* purine repressor with DNA. J. Bacteriol. 1997; 179:7394–7402. 9393704 10.1128/jb.179.23.7394-7402.1997 PMC179690

[57] Buescher J.M. , Liebermeister W., Jules M., Uhr M., Muntel J., Botella E., Hessling B., Kleijn R.J., Le Chat L., Lecointe F. et al. Global network reorganization during dynamic adaptations of *Bacillus subtilis* metabolism. Science. 2012; 335:1099–1103. 22383848 10.1126/science.1206871

[58] Sakai A. , Kita M., Katsuragi T., Ogasawara N., Tani Y. *yaaD* and *yaaE* are involved in vitamin B6 biosynthesis in *Bacillus**subtilis*. J. Biosci. Bioeng. 2002; 93:309–312. 16233205 10.1263/jbb.93.309

[59] Richts B. , Rosenberg J., Commichau F.M. A survey of pyridoxal 5′-phosphate-dependent proteins in the gram-positive model bacterium *Bacillus**subtilis*. Front. Mol. Biosci. 2019; 6:32. 31134210 10.3389/fmolb.2019.00032 PMC6522883

[60] Belitsky B.R. *Bacillus subtilis* GabR, a protein with DNA-binding and aminotransferase domains, is a PLP-dependent transcriptional regulator. J. Mol. Biol. 2004; 340:655–664. 15223311 10.1016/j.jmb.2004.05.020

[61] Belitsky B.R. , Sonenshein A.L. GabR, a member of a novel protein family, regulates the utilization of γ-aminobutyrate in *Bacillus**subtilis*. Mol. Microbiol. 2002; 45:569–583. 12123465 10.1046/j.1365-2958.2002.03036.x

[62] Hoffmann T. , Warmbold B., Smits S.H.J., Tschapek B., Ronzheimer S., Bashir A., Chen C., Rolbetzki A., Pittelkow M., Jebbar M. et al. Arsenobetaine: an ecophysiologically important organoarsenical confers cytoprotection against osmotic stress and growth temperature extremes. Environ. Microbiol. 2018; 20:305–323. 29159878 10.1111/1462-2920.13999

[63] Lee C.H. , Wu T.Y., Shaw G.C. Involvement of OpcR, a gbsr-type transcriptional regulator, in negative regulation of two evolutionarily closely related choline uptake genes in *bacillus**subtilis*. Microbiol. (United Kingdom). 2013; 159:2087–2096. 10.1099/mic.0.067074-0 23960087

[64] Maass S. , Sievers S., Zuhlke D., Kuzinski J., Sappa P.K., Muntel J., Hessling B., Bernhardt J., Sietmann R., Volker U. et al. Efficient, global-scale quantification of absolute protein amounts by integration of targeted mass spectrometry and two-dimensional gel-based proteomics. Anal. Chem. 2011; 83:2677–2684. 21395229 10.1021/ac1031836

[65] Maass S. , Wachlin G., Bernhardt J., Eymann C., Fromion V., Riedel K., Becher D., Hecker M. Highly precise quantification of protein molecules per cell during stress and starvation responses in *bacillus**subtilis*. Mol. Cell. Proteomics. 2014; 13:2260–2276. 24878497 10.1074/mcp.M113.035741 PMC4159648

[66] Rappu P. , Pullinen T., Mäntsälä P. In vivo effect of mutations at the PRPP binding site of the *Bacillus**subtilis* purine repressor. J. Bacteriol. 2003; 185:6728–6731. 14594850 10.1128/JB.185.22.6728-6731.2003 PMC262106

[67] Weng M. , Zalkin H. Mutations in the *Bacillus**subtilis* purine repressor that perturb PRPP effector function in vitro and in vivo. Curr.Microbiol. 2000; 41:56–59. 10919400 10.1007/s002840010091

[68] Taraban M. , Zhan H., Whitten A.E., Langley D.B., Matthews K.S., Swint-Kruse L., Trewhella J. Ligand-induced conformational changes and conformational dynamics in the solution structure of the lactose repressor protein. J. Mol. Biol. 2008; 376:466–481. 18164724 10.1016/j.jmb.2007.11.067 PMC2430094

[69] Zhang R.G. , Joachimiak A., Lawson C.L., Schevitz R.W., Otwinowski Z., Sigler P.B. The crystal structure of trp aporepressor at 1.8 Å shows how binding tryptophan enhances DNA affinity. Nature. 1987; 327:591–597. 3600756 10.1038/327591a0

[70] Zhao D. , Arrowsmith C.H., Jia X., Jardetzky O. Refined solution structures of the escherichia coli trp holo- and aporepressor. J. Mol. Biol. 1993; 229:735–746. 8433368 10.1006/jmbi.1993.1076

[71] Hickman J.W. , Harwood C.S. Identification of FleQ from *Pseudomonas aeruginosa* as a c-di-GMP-responsive transcription factor. Mol. Microbiol. 2008; 69:376–389. 18485075 10.1111/j.1365-2958.2008.06281.x PMC2612001

[72] Krasteva P.V. , Fong J.C.N., Shikuma N.J., Beyhan S., Navarro M.V.A.S., Yildiz F.H., Sondermann H. *Vibrio cholerae* VpsT regulates matrix production. Science. 2010; 327:866–868. 20150502 10.1126/science.1181185 PMC2828054

[73] Charity J.C. , Blalock L.A.T., Costante-Hamm M.M., Kasper D.L., Dove S.L. Small molecule control of virulence gene expression in francisella tularensis. PLoS Pathog. 2009; 5:e1000641. 19876386 10.1371/journal.ppat.1000641 PMC2763202

[74] Nano F.E. , Schmerk C. The *francisella* pathogenicity island. Ann. N. Y. Acad. Sci. 2007; 1105:122–137. 17395722 10.1196/annals.1409.000

[75] Belitsky B.R. , Sonenshein A.L. Genome-wide identification of *Bacillus**subtilis* cody-binding sites at single-nucleotide resolution. Proc. Natl Acad. Sci. U.S.A. 2013; 110:7026–7031. 23569278 10.1073/pnas.1300428110 PMC3637721

[76] Brinsmade S.R. , Alexander E.L., Livny J., Stettner A.I., Segrè D., Rhee K.Y., Sonenshein A.L. Hierarchical expression of genes controlled by the *Bacillus**subtilis* global regulatory protein codY. Proc. Natl. Acad. Sci. U.S.A. 2014; 111:2–7. 10.1073/pnas.1321308111 PMC4050614 24843172

[77] Sherlock M.E. , Sudarsan N., Breaker R.R. Riboswitches for the alarmone ppGpp expand the collection of RNA-based signaling systems. Proc. Natl. Acad. Sci. U.S.A. 2018; 115:6052–6057. 29784782 10.1073/pnas.1720406115 PMC6003355

[78] Wang B. , Dai P., Ding D., Del Rosario A., Grant R.A., Pentelute B.L., Laub M.T., Rosario A.Del, Grant R.A., Pentelute B.L. et al. Affinity-based capture and identification of protein effectors of the growth regulator ppGpp. Nat. Chem. Biol. 2018; 15:141–150. 30559427 10.1038/s41589-018-0183-4 PMC6366861

[79] Zhang Y. , Zbornikova E., Rejman D., Gerdes K. Novel (p)ppGpp binding and metabolizing proteins of *Escherichia**coli*. MBio. 2018; 9:e02188-17. 29511080 10.1128/mBio.02188-17 PMC5845004

[80] Buglino J. , Shen V., Hakimian P., Lima C.D. Structural and biochemical analysis of the obg GTP binding protein. Structure. 2002; 10:1581–1592. 12429099 10.1016/s0969-2126(02)00882-1

[81] Fan H. , Hahm J., Diggs S., Perry J.J.P., Blaha G. Structural and functional analysis of BipA, a regulator of virulence in enteropathogenic *Escherichia**coli*. J. Biol. Chem. 2015; 290:20856–20864. 26163516 10.1074/jbc.M115.659136 PMC4543647

[82] Kihira K. , Shimizu Y., Shomura Y., Shibata N., Kitamura M., Nakagawa A., Ueda T., Ochi K., Higuchi Y. Crystal structure analysis of the translation factor RF3 (release factor 3). FEBS Lett. 2012; 586:3705–3709. 22975312 10.1016/j.febslet.2012.08.029

[83] Pausch P. , Steinchen W., Wieland M., Klaus T., Altegoer F., Wilson D.N., Bange G. Structural basis for (p)ppGpp-mediated inhibition of the GTPase rbgA. J. Biol. Chem. 2018; 293:19699–19709. 30366986 10.1074/jbc.RA118.003070 PMC6314131

[84] DeLivron M.A. , Robinson V.L. *Salmonella enterica* serovar typhimurium BipA exhibits two distinct ribosome binding modes. J. Bacteriol. 2008; 190:5944–5952. 18621905 10.1128/JB.00763-08 PMC2519513

[85] Liu K. , Myers A.R., Pisithkul T., Claas K.R., Satyshur K.A., Amador-Noguez D., Keck J.L., Wang J.D. Molecular mechanism and evolution of guanylate kinase regulation by (p)ppGpp. Mol. Cell. 2015; 57:735–749. 25661490 10.1016/j.molcel.2014.12.037 PMC4336630

[86] Saraste M. , Sibbald P.R., Wittinghofer A. The P-loop - a common motif in ATP- and GTP-binding proteins. Trends Biochem. Sci. 1990; 15:430–434. 2126155 10.1016/0968-0004(90)90281-f

[87] Mandal M. , Boese B., Barrick J.E., Winkler W.C., Breaker R.R. Riboswitches control fundamental biochemical pathways in *Bacillus**subtilis* and other bacteria. Cell. 2003; 113:577–586. 12787499 10.1016/s0092-8674(03)00391-x

[88] Wang B. , Grant R.A., Laub M.T. ppGpp coordinates nucleotide and amino-acid synthesis in *E. coli* during starvation. Mol. Cell. 2020; 80:29–42. 32857952 10.1016/j.molcel.2020.08.005 PMC8362273

[89] Petchiappan A. , Gottesman S. How does the alarmone ppGpp change bacterial cell metabolism? From genome-wide approaches to structure to physiology. Mol. Cell. 2020; 80:1–2. 33007252 10.1016/j.molcel.2020.09.019

