# Supplementary material for: The nucleotide messenger (p)ppGpp is an anti-inducer of the purine synthesis transcription regulator PurR in Bacillus
Source: Nucleic Acids Res. 2021 Dec 30;50(2):847–66. doi: 10.1093/nar/gkab1281 (PMC8789054; doi:10.1093/nar/gkab1281)
Supplement: gkab1281_Supplemental_Files [file gkab1281_supplemental_files.zip › Supplementary Data revised.pdf]

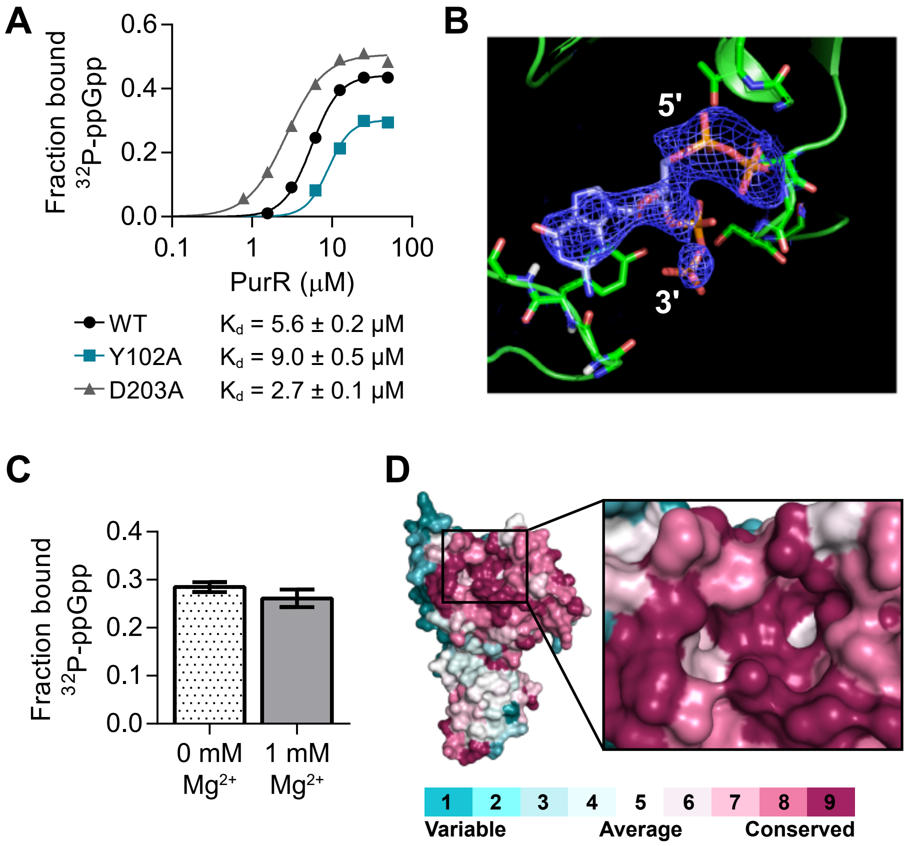

Figure S1. ppGpp binding to *B. subtilis* PurR.

A) DRaCALA binding curves of ^{32}P-ppGpp binding to Y102A and D203A variants of PurR. DRaCALA experiments in (A) and (C) were performed in technical triplicate. Error bars representing SEM may be shorter than the height of the symbols. K_d measurement is mean ± SEM. **B)** Omit electron density for ppGpp contoured to 2.4σ. The 5' and 3' arms of ppGpp are labeled. **C)** Mg^{2+} did not crystallize with PurR-ppGpp, and it is not necessary for the interaction as determined by DRaCALA. **D)** ConSurf analysis of 938 PurR proteins mapped onto PDB ID 1O57. Darker purple indicates more conserved residues. Inset shows the (p)ppGpp binding pocket.

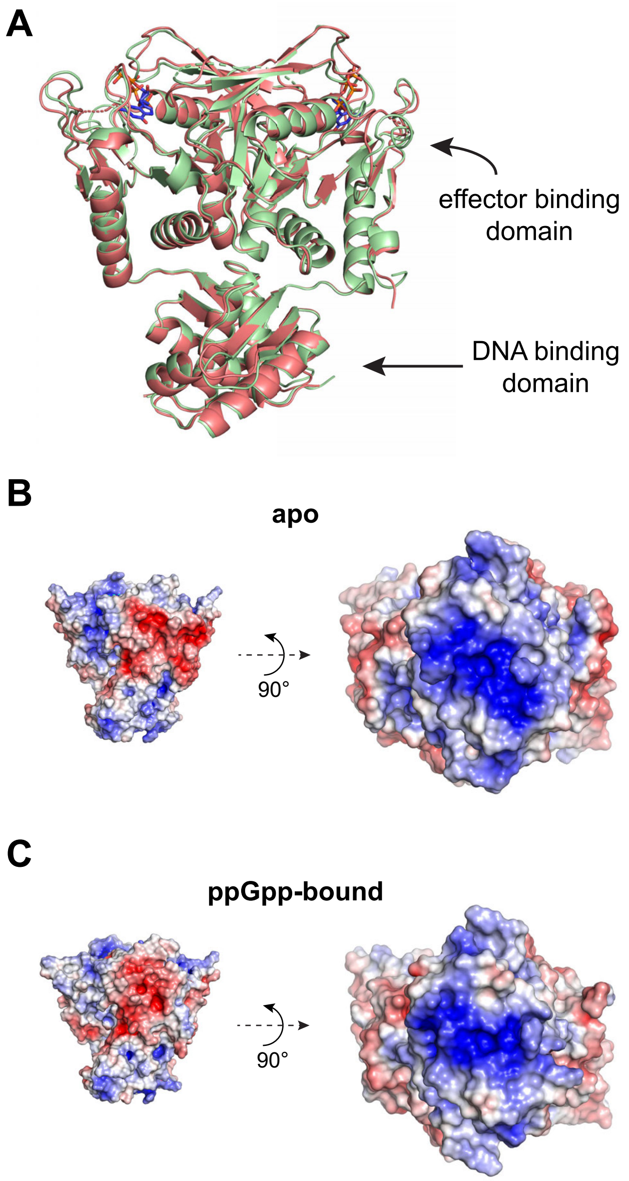

Figure S2. Effect of ppGpp on PurR conformation and electrostatics.

A) Overlay of *B. subtilis* apo PurR (green; PDB ID 1O57) and ppGpp-bound PurR (salmon) dimer. **B)** Poisson-Boltzmann continuum electrostatics of the DNA binding domain of apo PurR. **C)** Poisson-Boltzmann continuum electrostatics of the DNA binding domain of ppGpp-bound PurR. Scale of electrostatic potential is -5 (red) to +5 (blue) in all figures.

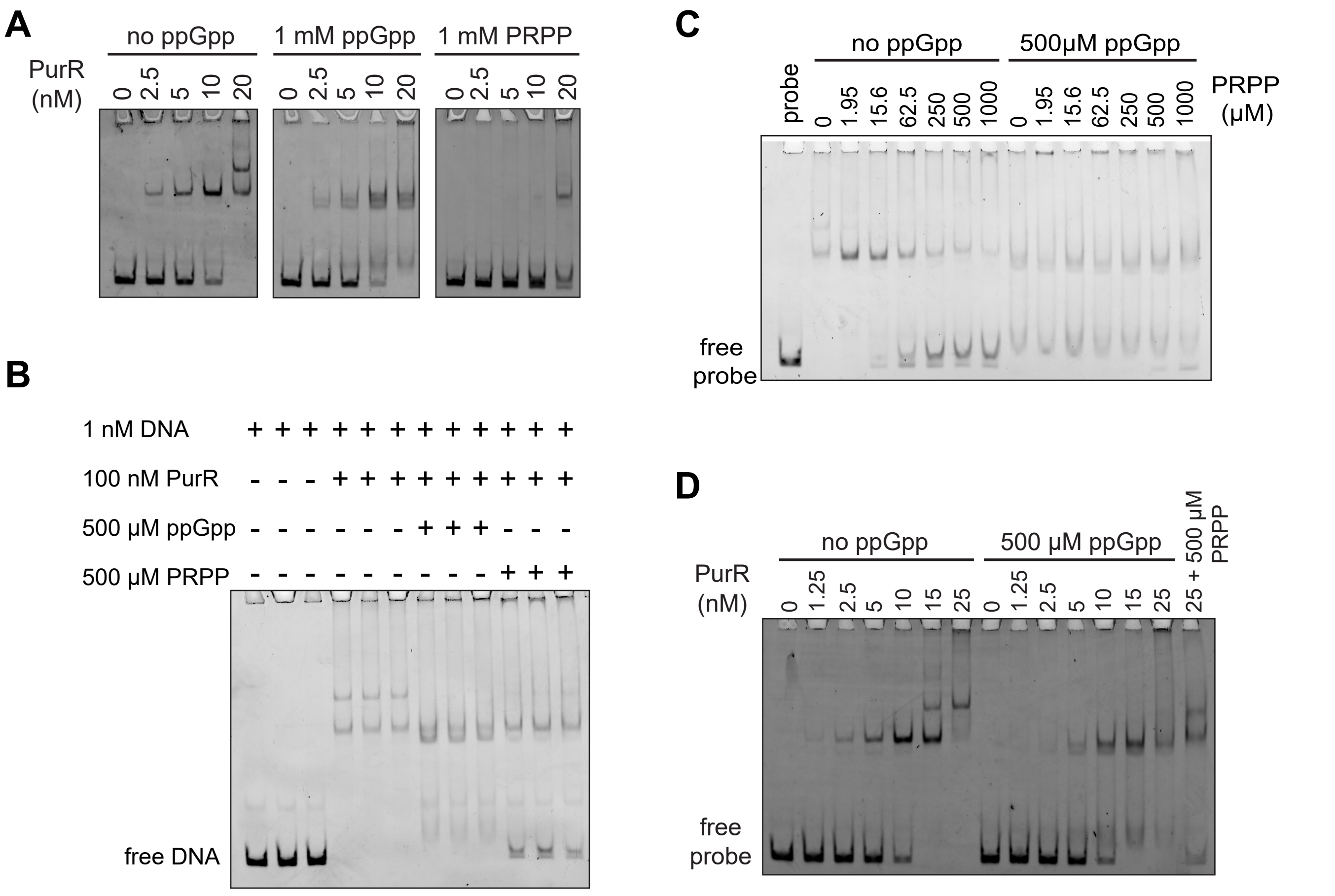

Figure S3. EMSAs of the effect of ppGpp and PRPP on PurR interaction with unlabeled DNA probe. EMSAs showing PurR interaction with an unlabeled 202 bp fragment upstream of the *pur* operon transcription start site. These EMSAs were performed at low KCl concentrations with no nonspecific DNA, and the probe was detected with nonspecific SYPRO Gold dye (see Materials and Methods). **A)** EMSAs showing PurR binding to DNA at increasing PurR concentrations in the presence of ppGpp or PRPP. **B)** EMSA showing the individual effects of ppGpp and PRPP on the binding of 100 nM PurR to DNA. **C)** EMSA showing the effect of ppGpp and PRPP competition on PurR binding to DNA at a PurR concentration of 100 nM. **D)** EMSA showing the effect of ppGpp alone on PurR interaction with DNA.

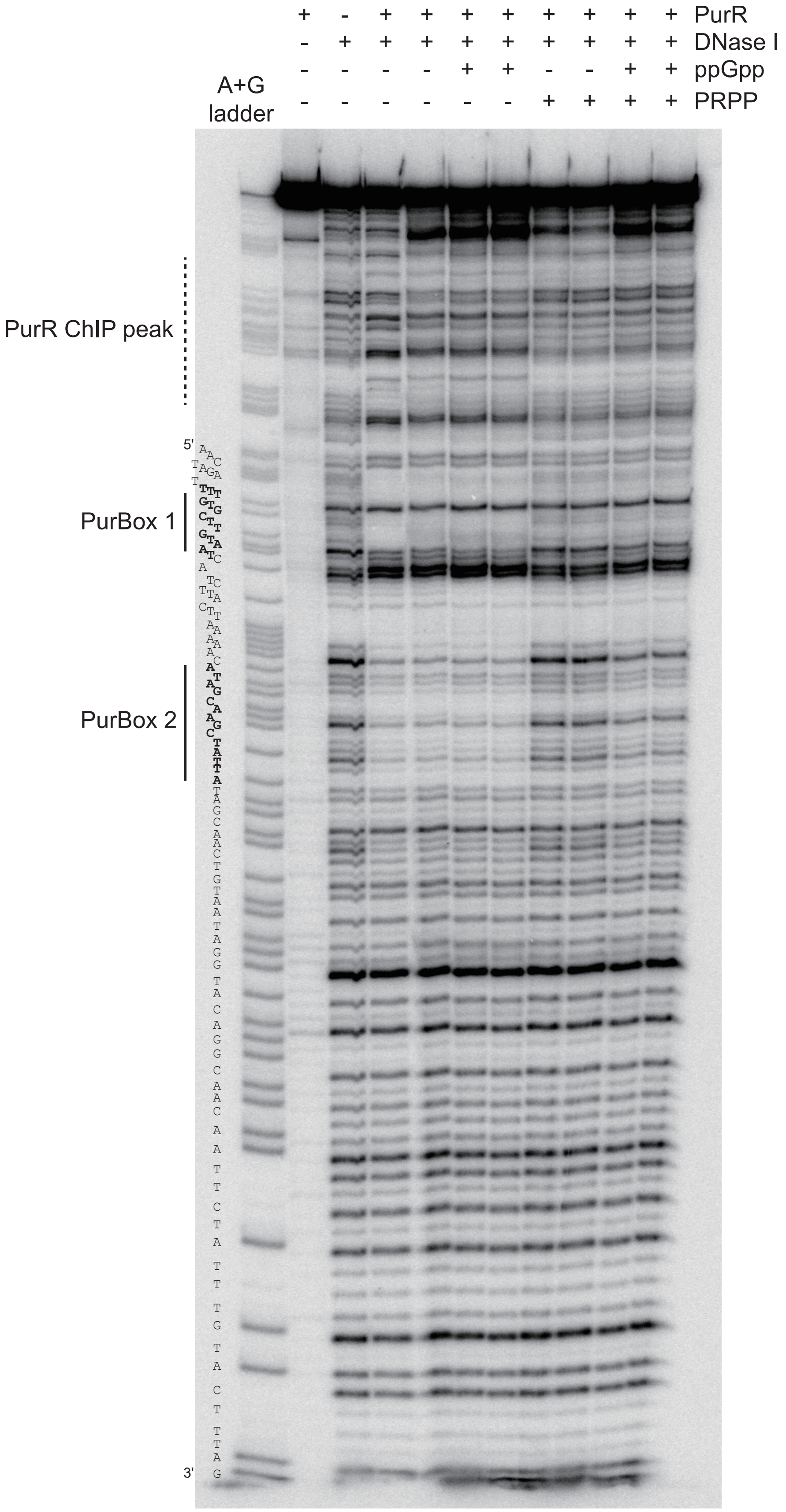

Figure S4. DNase I footprint of PurR-PurBox interaction, gel 1.
An uncropped gel image that was used in Figure 3E. ^{32}P-labeled PurBox fragment was incubated with or without PurR (50 nM), ppGpp (1 mM), and PRPP (16 μM), and then briefly digested by DNase I (3.2 μg/mL). The digestion product was analyzed by electrophoresis.

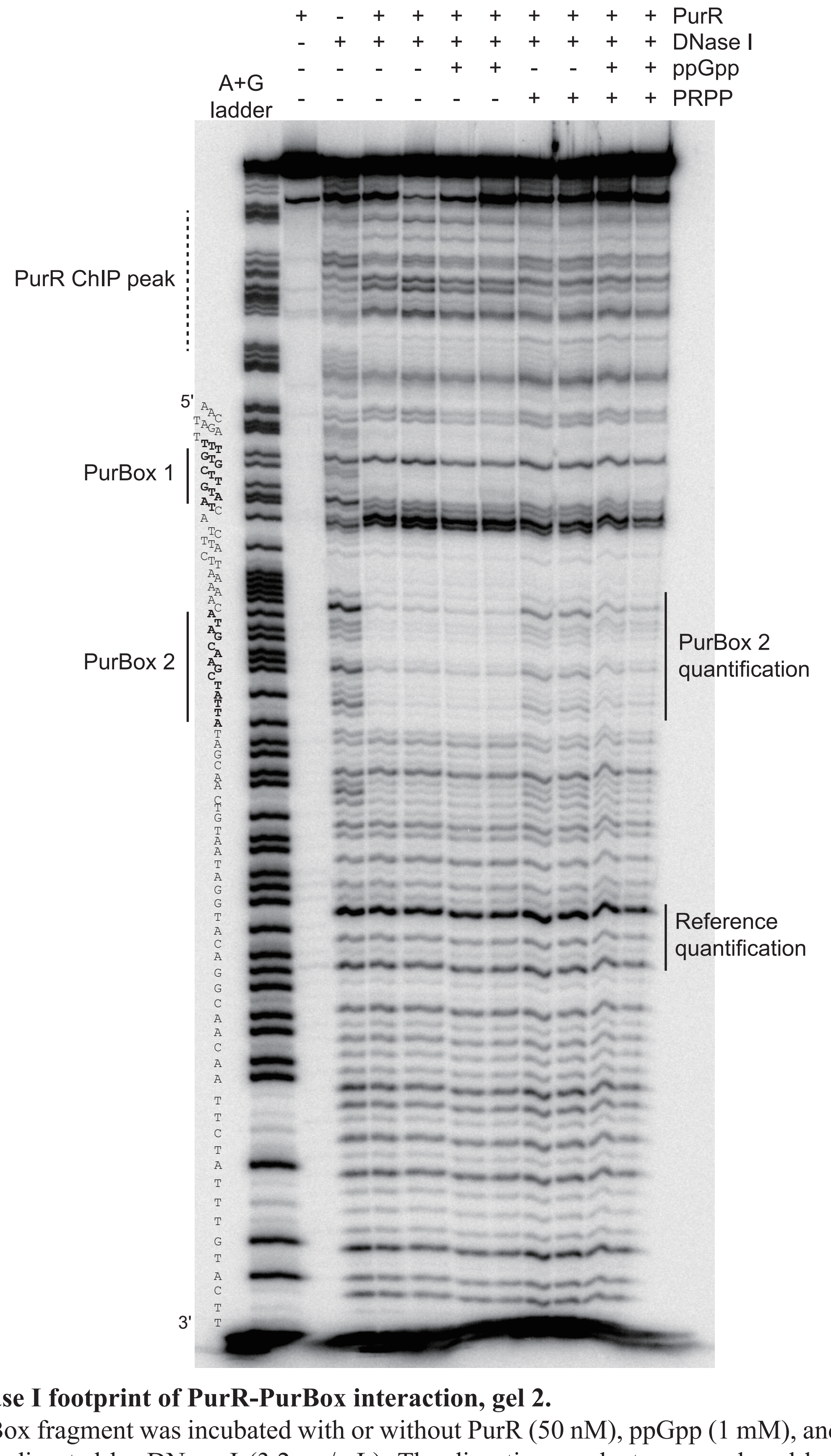

Figure S5. DNase I footprint of PurR-PurBox interaction, gel 2.
^{32}P-labeled PurBox fragment was incubated with or without PurR (50 nM), ppGpp (1 mM), and PRPP (16 μM), and then briefly digested by DNase I (3.2 μg/mL). The digestion product was analyzed by electrophoresis. Along with Figure S6, these are the raw data quantified in Figure 3F. Bands used for PurBox 2 quantification and normalization reference quantification are labeled on the right of the figure.

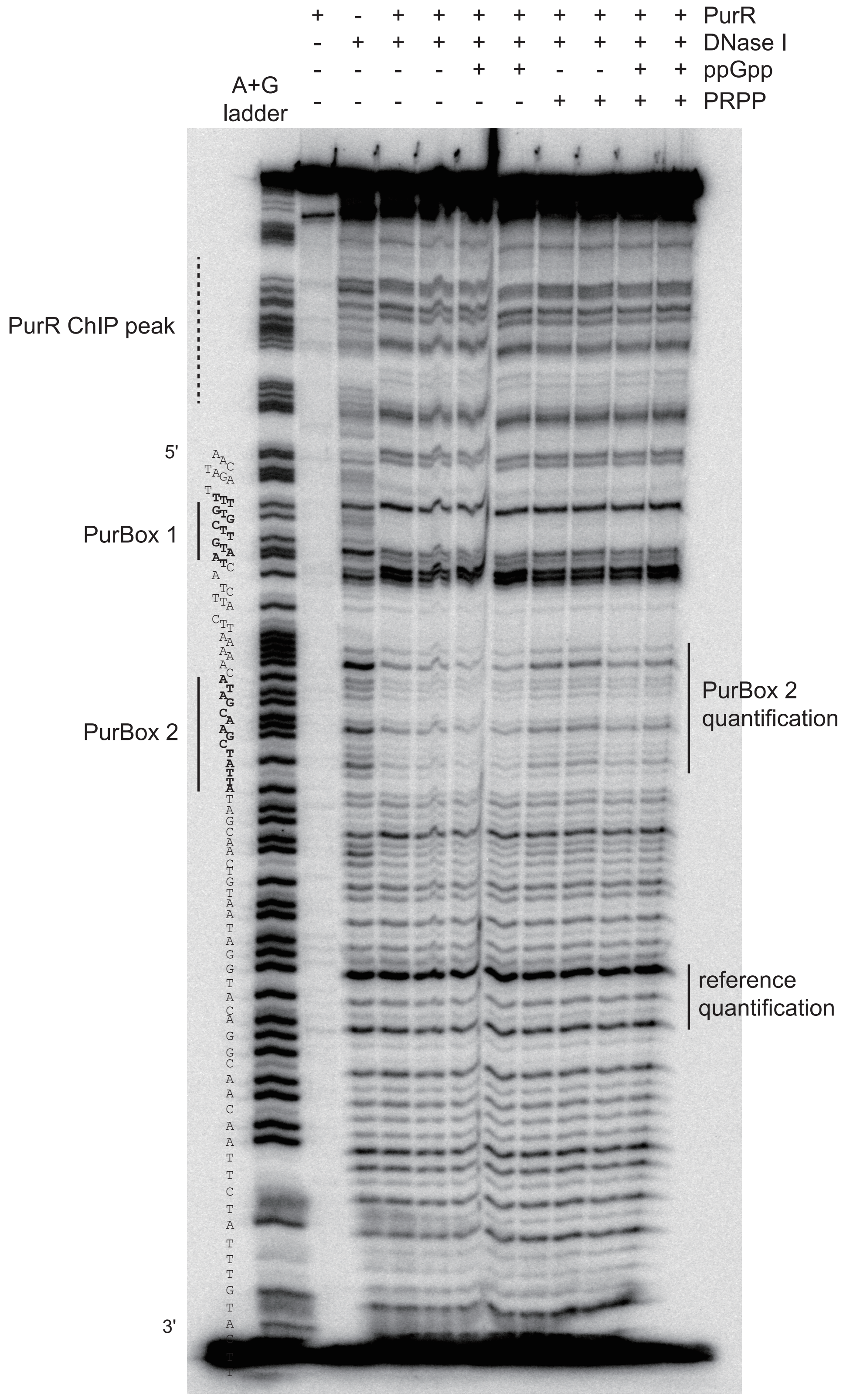

Figure S6. DNase I footprint of PurR-PurBox interaction, gel 3.
^{32}P-labeled PurBox fragment was incubated with or without PurR (50 nM), ppGpp (1 mM), and PRPP (16 μM), and then briefly digested by DNase I (3.2 μg/mL). The digestion product was analyzed by electrophoresis. Along with Figure S5, these are the raw data quantified in Figure 3F. Bands used for PurBox 2 quantification and normalization reference quantification are labeled on the right of the figure.

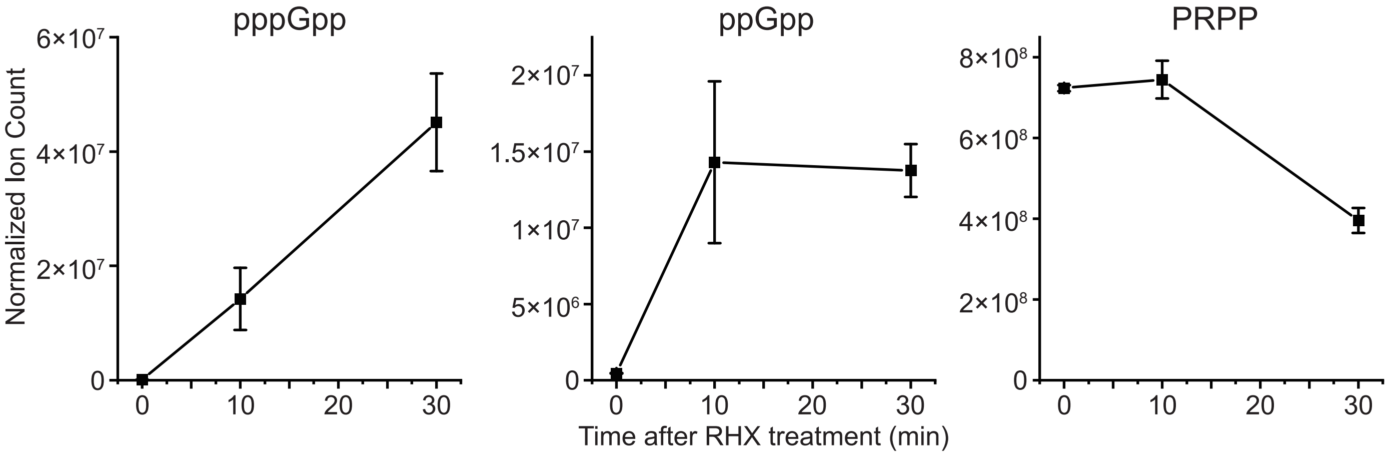

Figure S7. pppGpp, ppGpp and PRPP levels after arginine hydroxamate (RHX) treatment.
Metabolite levels are measured by LC-MS and normalized to unit volume and OD_{600} of the cultures. Error bars represent standard errors of the mean from two biological replicates.

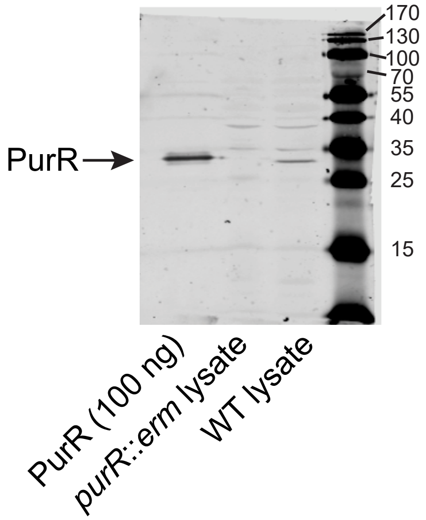

Figure S8. Western blot showing specificity of polyclonal anti-PurR antibody.

Western blot of PurR in wild type and *purR::ermHI B. subtilis*. Purified untagged *B. subtilis* PurR included as a control. Molecular weight markers in the ladder refer to kilodaltons.

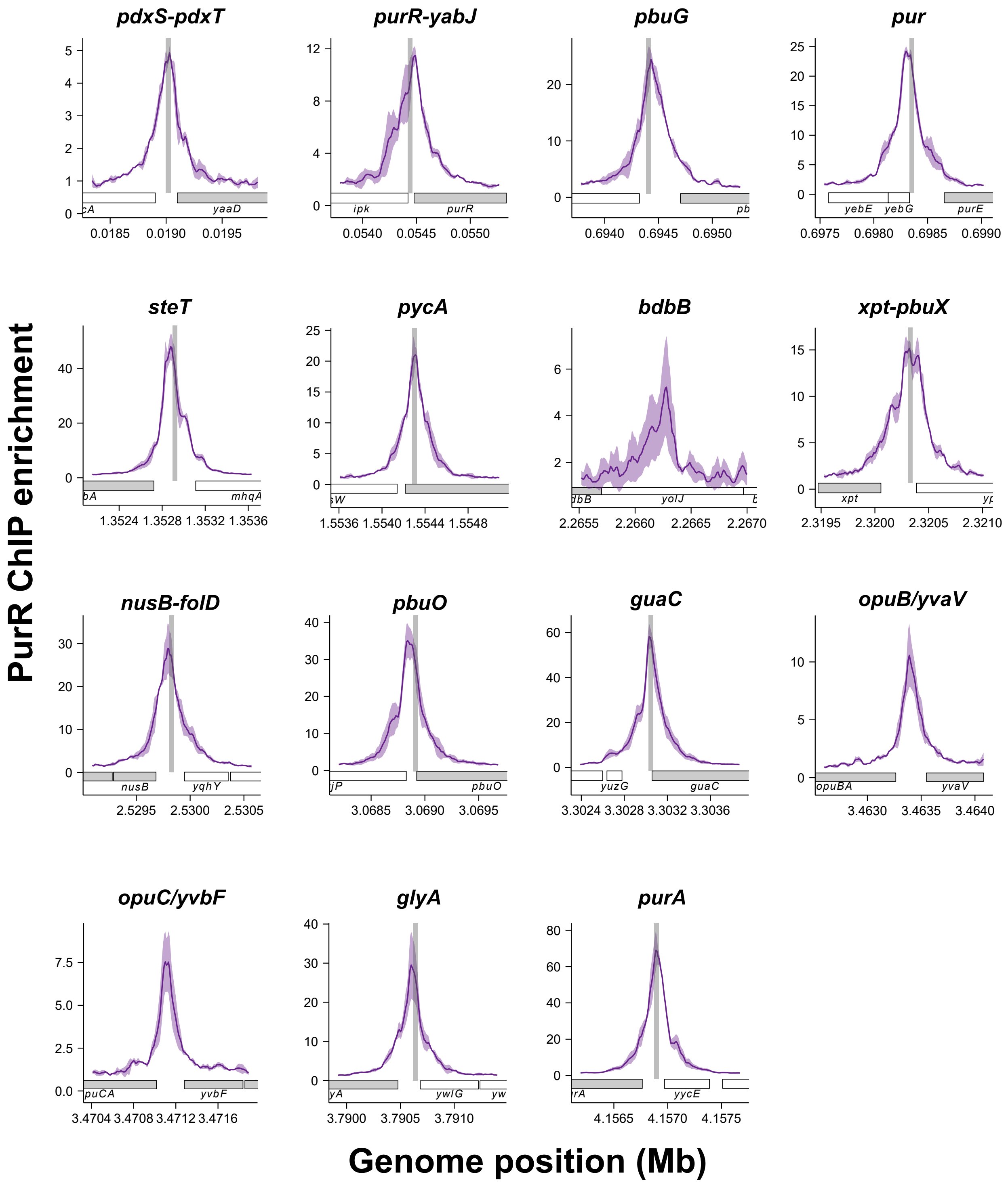

Figure S9. PurR binding sites during growth with nucleobases.

The 15 PurR ChIP peaks obtained during growth of *B. subtilis* with adenine, cytosine, guanine, and uracil nucleobases. Solid line represents mean of triplicate and shaded region represents standard deviation. Shaded gray vertical box represents the PurBox sequences identified at twelve of the PurR binding sites. Genes known or hypothesized to be regulated by PurR are colored gray. See Table 1 for more information.

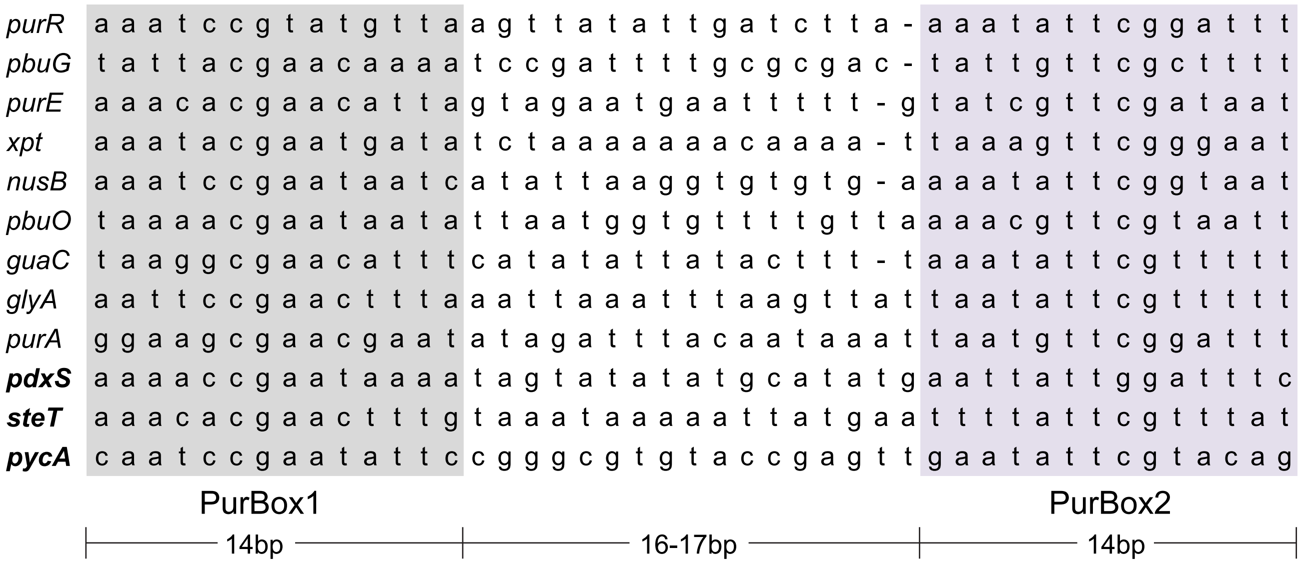

Figure S10. DNA sequences of the PurBoxes at 12 of the PurR binding sites.

DNA sequences of the PurBoxes at 12 of the 15 PurR binding sites. The remaining three sites have no apparent PurBox sequences. The PurBoxes are 14-bp inverted repeats separated by a 16 or 17-bp spacer. The PurBox sequences for *pdxS*, *steT*, and *pycA* (in bold) were identified in this study.

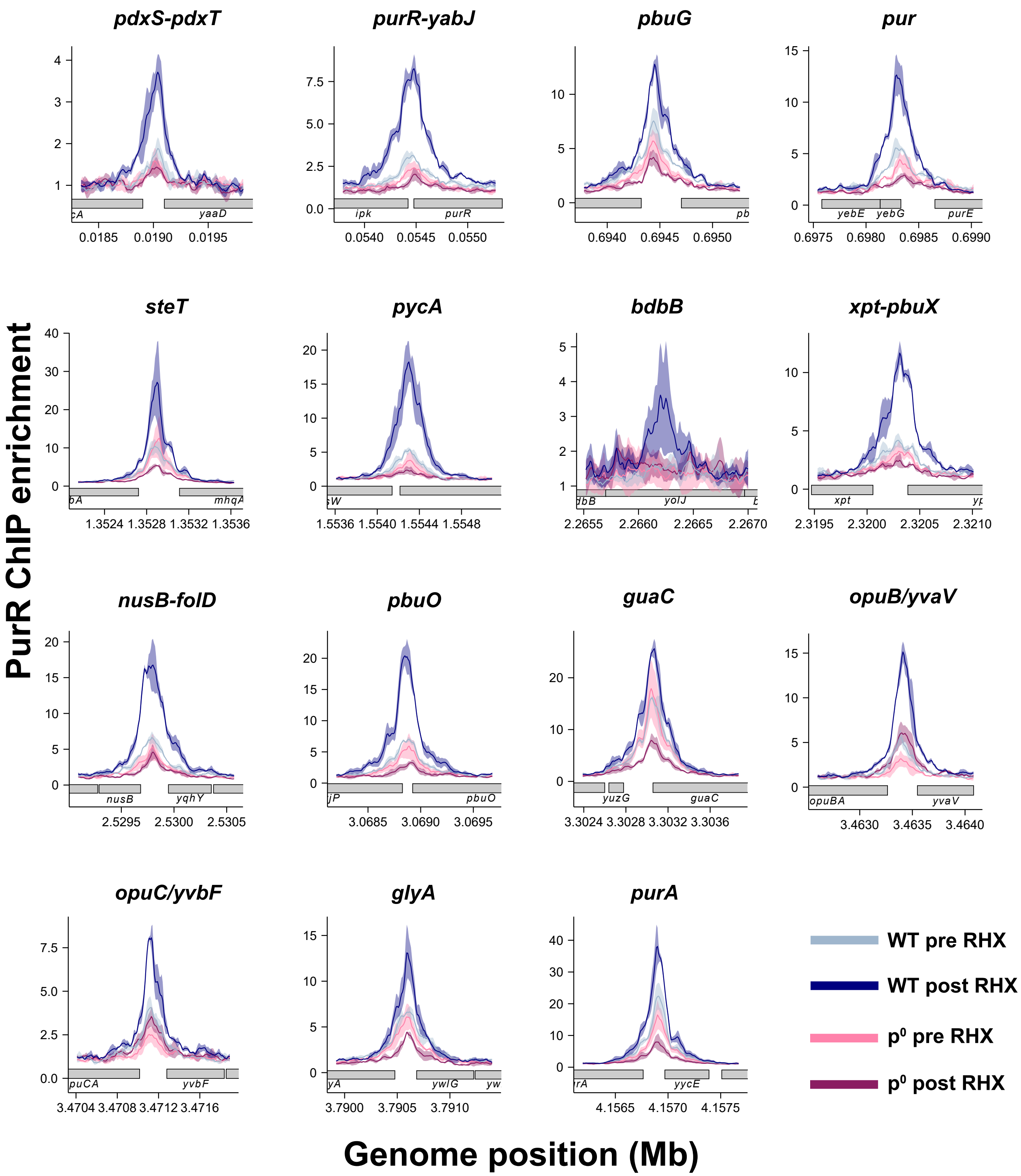

Figure S11. RHX treatment increases PurR enrichment at all 15 binding sites.
The 15 PurR binding sites determined by ChIP-seq before and after (p)ppGpp induction by arginine hydroxamate (RHX). Wild type (WT) before RHX is shown in cyan and after RHX is shown in blue. (p)ppGpp-null (p^0) before RHX is shown in pink and after RHX is shown in maroon. Solid line represents mean of triplicate and shaded region represents standard deviation. See Table 1 for more information.

AGAGCCTATTTTTATGGAAAAAAAGGAAAATGGGTCAATTCAGATCGTTCCGTGCG

PurR footprint

PurBox1

GGAAAAAAATCGTATTTGAAGGGAAATTGATCTAAAACACGAACATTAGTAGAATGA

TSS

PurBox2 -35 -10

ATTTTTGTATCGTTCGATAATATCGTTGACATTATCCATGTCCGTTGTTAAGATAAAC

ATGAAATCAAAACACGACCTCATATAATCTTGGGAATATGGCCCATAAGTTTCTACC

purine base riboswitch

CGGCAACCGTAAATTGCCGGACTATGCAGGAAAGTGATCGATAAAACTGACATGG

ATATATCGCAGAAGCGAACGACTGACGATACATGTACCATGCCCGGTTTGTATTGC

TTCCTCATAAGTGCAATGCAGAGCGGGTATTTTTTATTTTCTGAAAACAAAAGCATT

purE

AGAAGGTGGGGAACAGAATGCAGCCGCTAGTAGG...

Figure S12. DNA sequence of the regulatory region upstream of the *pur* operon in *B. subtilis*. The DNA sequence of 413 nt upstream of the *pur* operon, which starts with *purE* in *B. subtilis* (shaded in gray). The transcription start site (TSS) is shown by the arrow. The -10 and -35 promoter elements are marked with a line above the DNA sequence. PurBoxes 1 and 2 are annotated by shaded red boxes. The PurR DNase I footprint is underlined with a solid line. The footprint is from Ebbole and Zalkin (1989) *J. Biol. Chem.* **264**:3553-3561. The purine base-binding riboswitch is underlined with a dashed line.

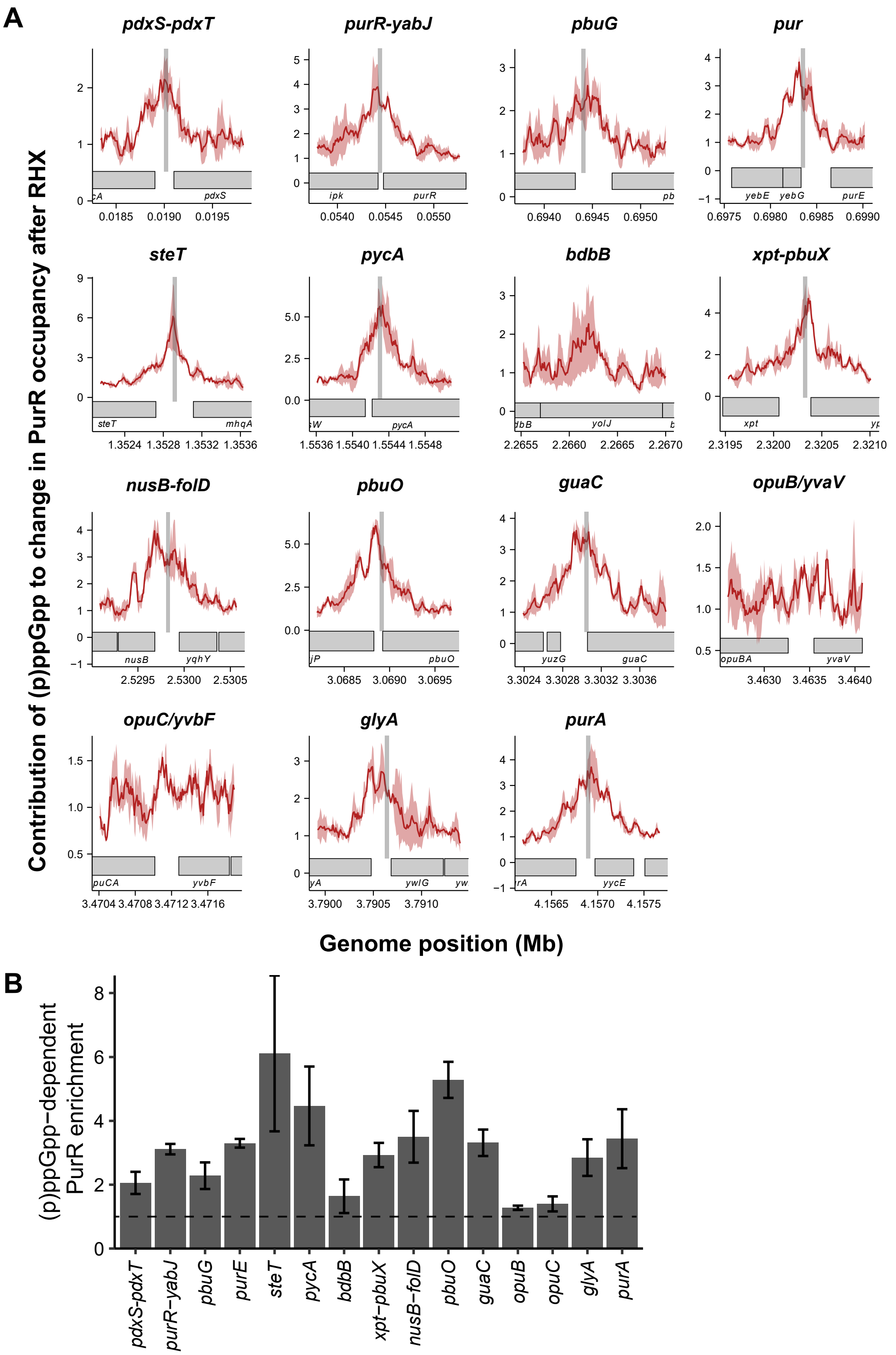

Figure S13. Contribution of (p)ppGpp to change in PurR occupancy.
A) The contribution of (p)ppGpp to change in PurR occupancy for each of the 15 PurR binding sites. The change in PurR occupancy after RHX treatment was calculated for WT and (p)ppGpp^0, and the ratio of the change in WT over (p)ppGpp^0 was used as the contribution of (p)ppGpp to PurR enrichment. The solid trace is the mean of biological triplicate and the shaded trace is the standard deviation. The vertical shaded bars mark the PurBoxes. **B)** The maximum point of each peak in (A) is plotted here, showing the maximum (p)ppGpp contribution for each binding site. The data are the mean of biological triplicate and the error bars are standard deviation. The horizontal dashed line at y=1 shows the expectation of no ppGpp influence on PurR enrichment.

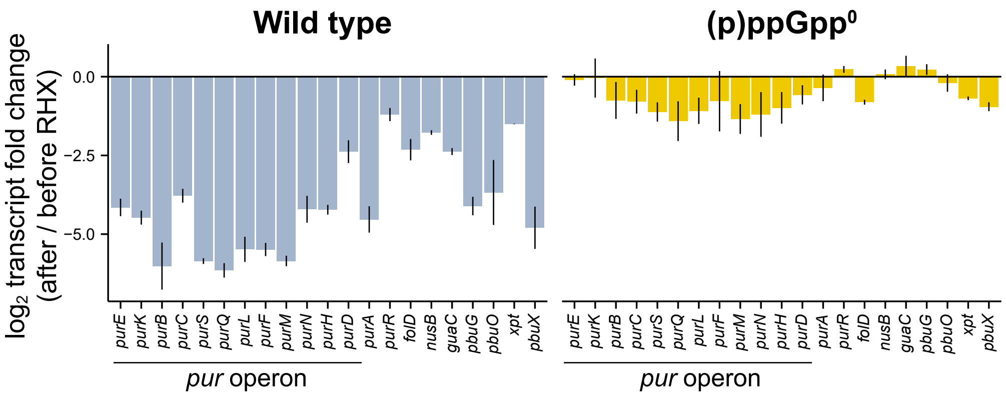

Figure S14. PurR-regulated genes are downregulated after RHX treatment in (p)ppGpp$^+$ *B. subtilis*. Change in transcript level of PurR-regulated genes after RHX treatment. These are the same data as those shown in Figure 5A with wild type and (p)ppGpp^0 *B. subtilis* plotted separately. These data are from Kriel et al. (2014). Mean of triplicate ± SD is shown.

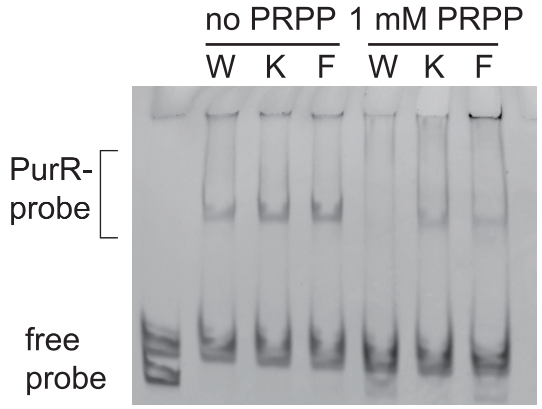

Figure S15. The ppGpp-refractory PurR variant can bind to the *pur* promoter and the binding is dissociated by PRPP.

Wild type PurR (W), $PurR^{Y102A/K207A}$ (K), and $PurR^{Y102A/F205A}$ (F) ppGpp-refractory variants binding to *pur* promoter are evaluated with EMSA with or without 1 mM PRPP. FAM-labeled 221 bp DNA probe: 5 nM; nonspecific DNA: 100 nM; PurR: 100 nM.

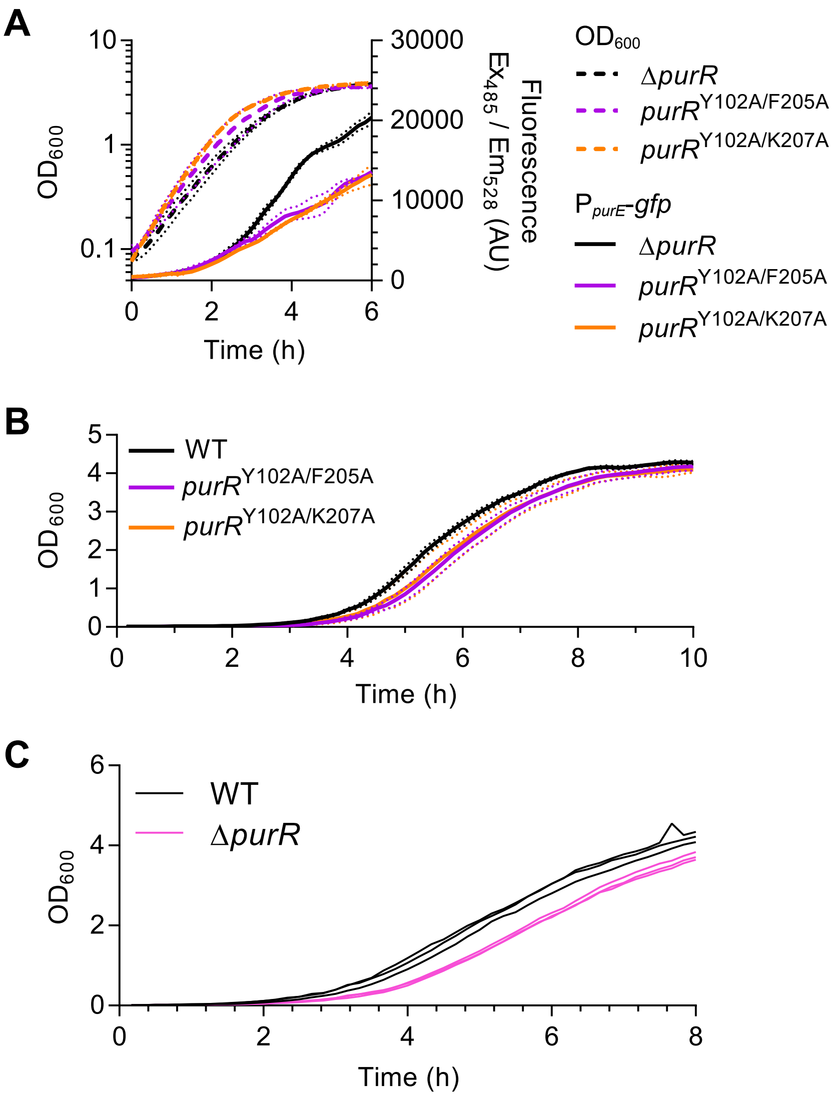

Figure S16. Dysregulated purine synthesis affects nutrient stress adaptation.

A) Expression of a P_{purE}-GFP reporter in Δ*purR*, *purR*$^{Y102A/F205A}$, and *purR*$^{Y102A/K207A}$ *B. subtilis* grown in Min+20aa medium. Dotted lines represent SEM of biological triplicate. **B)** Outgrowth of wild type, *purR*$^{Y102A/F205A}$, and *purR*$^{Y102A/K207A}$ in Min+20aa media following a transient downshift from Min+20aa to Min before being resuspended in Min+20aa. Error (dotted lines) is SEM of biological triplicate. **C)** Growth of wild type and Δ*purR* in Min+20aa following a transient nutrient downshift from Min+20aa to Min medium and subsequent upshift back to Min+20aa. Individual traces of three biological replicates are shown.

SUPPLEMENTARY TABLES

Table S1. pppGpp targets identified by screening His- and HisMBP-tagged ORFeome libraries of *B. anthracis*.

**Locus ID**	**Description**	**Z-score His**	**Z-score HisMBP**	**Z-score His+GTP**	**Z-score HisMBP+GTP**
BA3162	5'-nucleotidase, putative GMP -> guanosine	6.27	5.37	1.73	0.54
BA4322	5'-nucleotidase family protein	5.47	-2.28	2.56	-2.41
BA4672	spo0B-associated GTP-binding protein (Obg)	4.31	4.95	3.66	4.09
BA4455	membrane protein, putative	4.23	0.16	1.31	1.68
BA1525	GTPase family protein (Der. EngA)	3.97	1.38	4.17	4.53
BA0063	hypoxanthine phosphoribosyltransferase (Hpt-1)	3.92	3.05	7.33	6.23
BA4788	hypothetical protein 152 aa with heme-binding site, iron transfer operon	3.52	1.47	1.43	-0.28
BA5734	tRNA modification GTPase TrmE	3.27	2.47	2.82	3.44
BA4098	hypothetical protein YuaY	3.24	1.26	0.53	0.27
BA2853	cell division protein DivIC	3.09	2.44	0.65	1.58
pxo1_47	hypothetical protein pxo1_47	3.05	-0.07	0.4	0.74
BA4915	acetyl-CoA synthetase	2.98	0.33	0.22	1.19
BA1591	xanthine phosphoribosyltransferase	2.83	6.18	6.12	7.71
BA1692	hypothetical protein	2.77	1.43	0.86	1.07
BA5074	hypoxanthine phosphoribosyltransferase (Hpt-2)	2.72	2.05	4.49	3.38
BA5716	adenylosuccinate synthetase purA	2.69	2.32	4.25	3.67
BA1998	NH(3)-dependent NAD(+) synthetase (NadE)	2.55	0.38	0.6	0.7
BA3589	hypothetical protein	2.53	0.38	0.48	0.87
BA3846	PTS system, fructose-specific IIABC component	2.5	-0.23	0.07	-0.25
BA1925	cobalamin synthesis protein/P47K family protein	0.75	3.87	2.58	2.53
BA5472	D-amino acid aminotransferase	0.79	3.79	-1.6	1.46
BA1531	DNA-binding protein HU	1.39	3.74	1.2	-0.09
BA0692	hypothetical protein	0.99	3.55	1.67	1.69
BA1828	GTP-binding protein HflX	2.3	3.51	2.09	3.27
BA4699	transcriptional regulator, MarR family	0.41	3.33	-1.62	-1.85
BA0596	nicotinate phosphoribosyltransferase, putative	1.58	3.32	-0.18	0.87
BA1407	hypothetical protein	0.21	3.26	0.76	0
BA1212	hypothetical protein (YjbM)	1.49	3.24	0.56	6.69
pxo1_60	hypothetical protein pxo1_60	2.16	3.21	0.78	0.39
BA3140	hypothetical protein	0.29	3.15	0.34	-0.3
BA0150	polysaccharide deacetylase, putative	-0.79	2.98	-0.02	2.11
BA2932	glutathionylspermidine synthase, putative	0.35	2.93	-0.11	-1.01
BA0306	DNA ligase, NAD-dependent	1.33	2.91	1.2	0.17
BA2254	hypothetical protein	1.17	2.88	1.12	-0.09
BA4700	organic hydroperoxide resistance protein	0.51	2.85	1.02	1.47
BA0880	hypothetical protein	0.87	2.84	0.61	0.17
BA2278	hypothetical protein	1.12	2.83	1.72	-1.08
BA0721	hypothetical protein	-0.77	2.76	-0.59	1.25
BA3131	alcohol dehydrogenase, zinc-containing	0.77	2.76	1.08	1.76
BA5251	hypothetical protein	0.04	2.7	0.49	-0.26
BA2837	hypothetical protein	0.59	2.68	0.78	1.11
BA2636	sensor histidine kinase	0.57	2.6	0.36	0.38
BA2470	hypothetical protein	1.91	2.59	-0.99	0.44
BA1349	hypothetical protein	1.24	2.57	0.97	1.8
BA4397	DNA repair protein RecN	0.02	2.57	1.16	0.64
BA2163	HD domain protein	0.56	2.55	0.79	1.49
BA2625	hypothetical protein	-1.46	2.53	-0.62	0.14

BXB0074	hypothetical protein BXB0074	1.26	2.52	0.8	1.24
BA1267	hypothetical protein	0.23	2.51	-1.32	1.96
BA4807	HD domain protein	-0.04	2.5	-0.73	0.57
BA0044	pur operon repressor PurR	2.39	1.31	5.02	5.27
BA3029	succinylornithine transaminase, putative	1.49	-3.78	4.7	-2.21
BA4524	GTP-binding protein Era	2.11	0.61	4.49	1.52
BA4009	guanylate kinase, putative (Gmk)	-3.51	0.83	3.56	2.21
BA1257	hypothetical protein	1.48	1.93	3.23	2.24
BA4004	methionyl-tRNA formyltransferase (Fmt)	1.76	1.4	3.03	-0.09
BA5189	hypothetical protein	2.35	1.69	2.99	3.59
BXB0111	hypothetical protein BXB0111	0.99	-0.69	2.83	0.46
BA5043	mutT/nudix family protein	-1.95	-2.35	2.83	4.33
BA4571	nitroreductase family protein	1.76	-0.24	2.76	-0.36
BA4637	GTP pyrophosphokinase (RelA)	2.32	1.79	2.67	1.83
BA0272	sensor histidine kinase	1.87	-0.66	2.64	-0.89
BA1917	rrf2 family protein	-4.09	-0.08	2.54	0.88
BA3435	alcohol dehydrogenase, zinc-containing	-0.18	-0.21	-0.45	3.78
BA3458	transcriptional regulator, GntR family	0.79	0.69	0.45	3.06
BA2105	hypothetical protein	0.14	0.07	-0.04	2.87
BA0016	isochorismatase family protein	-1.35	1.84	-1.6	2.75
BA2452	hypothetical protein	0.66	-1.59	2.1	2.71
BA1974	lipoprotein, putative	-0.85	-0.02	0.45	2.56
BA0445	prophage LambdaBa04, transactivating regulatory domain protein	-0.07	1.34	0.27	2.54

Putative pppGpp targets identified by screening His- and HisMBP-tagged *B. anthracis* ORFeome libraries with or without non-radiolabeled GTP. The fraction of ^{32}P-pppGpp binding was normalized as a Z-score (number of standard deviations from the mean). Proteins with a Z-score greater than 2.5σ in any of the four screens are listed here. Z-scores greater than 2.5σ are shaded. PurR (BA0044) is in bold. The His and HisMBP screens were previously reported in Yang *et al.* (2020).

Table S2. Data collection and refinement statistics for *B. subtilis* PurR-ppGpp structure.

PDB code	7RMW
Space group	P1
Cell constants (Å)	a=60.26, b=90.97, c=98.71
Cell angles (º)	α=62.75, β=75.35, γ=78.53
Resolution (Å)	80.50-2.45
R_{sym}(%)[a]	5.5 (37.7)[b]
R_{pim} (%)	3.9 (27.7)
Overall I/□(I)	7.8 (2.3)
#Unique Reflections	62863
#Total Reflections	199456
% Complete	95.2 (91.3)
CC(1/2)	0.997 (0.940)
Multiplicity	2.6 (2.4)
Refinement Statistics	
Resolution (Å)	80.50-2.45
R_{work}/R_{free}(%)[c]	19.4/23.9
Rmsd	
Bond angles (º)	0.963
Bond lengths (Å)	0.007
Ramachandran analysis	
Favored (%/)	96.1
Disallowed(%)	0.0

[a]R_{sym} = ΣΣ|Ihkl-Ihkl(j)|/ ΣIhkl, where Ihkl(j) is observed intensity and Ihkl is the final average value of intensity. [b]values in parentheses are for the highest resolution shell. [c]R_{work} = Σ||Fobs| - |Fcalc||/Σ|Fobs| and R_{free} = Σ||Fobs| - |Fcalc||/Σ|Fobs|; where all reflections belong to a test set of 5% randomly selected data.

Table S3. List of primers.

Primer ID	Sequence (5' to 3')	Purpose
oJW492	GCTTTGTTAGCAGCCGGATCAG	amplify pLIC-trPC-HA insert
oJW1124	CCGCACCTGTGGCGCCGGTG	amplify pLIC-trPC-HA insert
oJW902	AAAGAGGCGCTTTTGACGTG	amplify *relA*::*mls*
oJW903	TTGTTGACCCGGGACATGGA	amplify *relA*::*mls*
oJW1285	AATGGAAGAGCCTATTTTTATG	amplify 202bp PurR binding region upstream of *pur* operon for EMSA
oJW1286	ATATGAGGTCGTGTTTTGATTTC	amplify 202bp PurR binding region upstream of *pur* operon for EMSA
oJW2775	GACGGTCTCAGCTGGCTGTAGG CATAGGCTTGGTTATG	amplify pJW557 for Golden Gate assembly
oJW2821	GTAGGTCTCTAAGGATTTCGCG GGATCGAGATCCTGCATTAATG	amplify pJW557 for Golden Gate assembly
oJW2842	TACTTCCAATCCAATGCAATGA AGTTTCGTCGCAGCG	amplify *B. subtilis* PurR for LIC cloning
oJW2843	TTATCCACTTCCAATGTTATTAT GATTCTGTCTCTCCATTCTTTAA AAGATT	amplify *B. subtilis* PurR for LIC cloning
oJW3384	TACTTCCAATCCAATGCAATGG ATAAATTTACAAGAAATCAACG	amplify *C. sporogenes* PurR for LIC cloning
oJW3385	TTATCCACTTCCAATGTTATTAT TATCTGAATAATTTAGATGGCT TAACTAC	amplify *C. sporogenes* PurR for LIC cloning
oJW3386	TACTTCCAATCCAATGCATTGA AAATTCGGAGAAGTGAAC	amplify *E. faecalis* PurR for LIC cloning
oJW3387	TTATCCACTTCCAATGTTATTAT TATTTCTCTGTGAAATAGTTTCC TG	amplify *E. faecalis* PurR for LIC cloning
oJW3394	TACTTCCAATCCAATGCAATGA AATTGCGACGTAGCG	amplify *S. mutans* PurR for LIC cloning
oJW3395	TTATCCACTTCCAATGTTATTAT CATTTACTAAAAATATTTCCCA ACTTAAC	amplify *S. mutans* PurR for LIC cloning
oJW3616	CTTCCGGGCGGTGCGGTATATT TAAC	megaprimer SDM *B. subtilis* Y102A
oJW3413	CATTATTGATGACGCTATGAAA GCAGGC	megaprimer SDM *B. subtilis* F205A
oJW3414	GATGACTTTATGGCAGCAGGCG G	megaprimer SDM *B. subtilis* K207A
oJW3398	CTATGGCGGAACTGCATTAGC	amplify *purR::ermHI* for transformation into NCIB3610
oJW3399	CGATTTTTTCTGAAATCCTGCTC G	amplify *purR::ermHI* for transformation into NCIB3610

oJW3438	GCTTTGTTAGCAGCCGGATCAG	amplify 500bp upstream of *pur* operon for cloning into pJW669
oJW3439	CCGCACCTGTGGCGCCGGTG	amplify 500bp upstream of *pur* operon for cloning into pJW669
oJW3426	GACGGTCTCAAAACTCCCGCCA CATTTGCGTTAAACTCATCCAA CGTTTTAGAGACCGTC	guide RNA for *purR(D203A)* (pJW692), *purR(F205A)* (pJW719), and *purR(K207A)* (pJW720)
oJW3427	GACGGTCTCTAAAACGTTGGAT GAGTTTAACGCAAATGTGGCGG GAGTTTTGAGACCGTC	guide RNA for *purR(D203A)* (pJW692), *purR(F205A)* (pJW719), and *purR(K207A)* (pJW720)
oJW3752	GACGGTCTCAAAACGCAAACAC GGAAGCAAACAGCTTCCCTACC TGTTTTAGAGACCGTC	guide RNA for *purR(Y102A)* (pJW710)
oJW3753	GACGGTCTCTAAAACAGGTAGG GAAGCTGTTTGCTTCCGTGTTTG CGTTTTGAGACCGTC	guide RNA for *purR(Y102A)* (pJW710)
oJW4028	/56-FAM/ATTATATGAGGTCGTGTTT TGATTTCA	amplify FAM-labeled 221bp PurR binding region upstream of *pur* operon for EMSA
oJW4029	ATCTGCCTGTAAGGCGAATGG	amplify FAM-labeled 221bp PurR binding region upstream of *pur* operon for EMSA
oJW4280	GGTGGTAAGCTTATCTGCCTGT AAGGCGAATGG	amplify PurBox DNA to construct probe for DNase I footprinting
oJW4281	GGTGGTGGATCCGATTATATGA GGTCGTGTTTTGATTTCA	amplify PurBox DNA to construct probe for DNase I footprinting
	CAGGGTCTCACCTTTTGCAGAG CGCGAAATTGATGTTGTCATGA CCGTTGCCACGAAAGGCATCCC TCTTGCGTACGCAGCTGCAAGC TATTTGAATGTGCCTGTTGTGAT CGTTCGTAAAGACAATAAGGTA ACAGAGGGCTCCACAGTCAGCA TTAATTACGTTTCAGGCTCCTCA AACCGCATTCAAACAATGTCAC TTGCGAAAAGAAGCATGAAAA CGGGTTCAAACGTACTCATTAT TGCGGACTTTATGAAAGCAGGC GGCACCATTAATGGTATGATTA ATCTGTTGGATGAGTTTAACGC AAATGTGGCGGGAATCGGCGTC TTAGTTGAAGCCGAAGGAGTAG ATGAACGTCTTGTTGACGAATA TATGTCACTTCTTACTCTTTCAA CCATCAACATGAAAGAGAAGTC CATTGAAATTCAGAATGGCAAT	Repair template for *purR(D203A)* (pJW692)

	TTTCTGCGTTTTTTTAAAGACAA TCTTTTAAAGAATGGAGAGACG CTGTGAGACCGAC	
	CAGGGTCTCACCTTATACCGCT AACCTTTTTCTCTGAGCGGTAT GAATCTGCAAAATCATCGATCA GTGAAGATTTAACAATTATTAA ACAAACCTTTGAACAGCAGGGG ATTGGTACTTTGCTTACTGTTCC CGGAGCTGCCGGAGGCGTTAAA TATATTCCGAAAATGAAGCAGG CTGAAGCTGAAGAGTTTGTGCA GACACTTGGACAGTCGCTGGCA AATCCTGAGCGTATCCTTCCGG GCGGTGCGGTATATTTAACGGA TATCTTAGGAAAGCCATCTGTA CTCAGTAAGGTAGGGAAGCTGT TTGCTTCCGTGTTTGCAGAGCG CGAAATTGATGTTGTCATGACC GTTGCCACGAAAGGCATCCCTC TTGCGTACGCAGCTGCAAGCTA TTTGAATGTGCCTGTTGTGATC GTTCGTAAAGACAATAAGGTAA CAGAGGGCTCCACAGTCAGCAT TAATTACGTTTCAGGCTCCTCA AGCTGTGAGACCGAC	Repair template for *purR(Y102A)* (pJW710)
	CAGGGTCTCACCTTTTGCAGAG CGCGAAATTGATGTTGTCATGA CCGTTGCCACGAAAGGCATCCC TCTTGCGTACGCAGCTGCAAGC TATTTGAATGTGCCTGTTGTGAT CGTTCGTAAAGACAATAAGGTA ACAGAGGGCTCCACAGTCAGCA TTAATTACGTTTCAGGCTCCTCA AACCGCATTCAAACAATGTCAC TTGCGAAAAGAAGCATGAAAA CGGGTTCAAACGTACTCATTAT TGACGACGCGATGAAAGCAGG CGGCACCATTAATGGTATGATT AATCTGTTGGATGAGTTTAACG CAAATGTGGCGGGAATCGGCGT CTTAGTTGAAGCCGAAGGAGTA GATGAACGTCTTGTTGACGAAT ATATGTCACTTCTTACTCTTTCA ACCATCAACATGAAAGAGAAG TCCATTGAAATTCAGAATGGCA	Repair template for *purR(F205A)* (pJW719)

	ATTTTCTGCGTTTTTTTAAAGAC<br>AATCTTTTAAAGAATGGAGAGA<br>CGCTGTGAGACCGAC	
	CAGGGTCTCACCTTTTGCAGAG<br>CGCGAAATTGATGTTGTCATGA<br>CCGTTGCCACGAAAGGCATCCC<br>TCTTGCGTACGCAGCTGCAAGC<br>TATTTGAATGTGCCTGTTGTGAT<br>CGTTCGTAAAGACAATAAGGTA<br>ACAGAGGGCTCCACAGTCAGCA<br>TTAATTACGTTTCAGGCTCCTCA<br>AACCGCATTCAAACAATGTCAC<br>TTGCGAAAAGAAGCATGAAAA<br>CGGGTTCAAACGTACTCATTAT<br>TGACGACTTTATGGCGGCAGGC<br>GGCACCATTAATGGTATGATTA<br>ATCTGTTGGATGAGTTTAACGC<br>AAATGTGGCGGGAATCGGCGTC<br>TTAGTTGAAGCCGAAGGAGTAG<br>ATGAACGTCTTGTTGACGAATA<br>TATGTCACTTCTTACTCTTTCAA<br>CCATCAACATGAAAGAGAAGTC<br>CATTGAAATTCAGAATGGCAAT<br>TTTCTGCGTTTTTTTAAAGACAA<br>TCTTTTAAAGAATGGAGAGACG<br>CTGTGAGACCGAC	Repair template for *purR(K207A)* (pJW720)

Table S4. List of plasmids.

**Plasmid ID**	**Construct**	**Purpose**	**Reference**
pJW270	pLIC-trPC-HMA	Expression vector	PMID: 12071693
pJW664	pLIC-trPC-HMA *B. subtilis purR*	Expression of HisMBP-tagged *B. subtilis* PurR	this work
pJW669	pPB41 with promoterless superfolder GFP	Cloning promoter of interest controlling sfGFP expression	this work
pJW686	pJW669 P_{purE}-sfGFP	GFP reporter for P_{purE} operon, for recombination at *amyE*	this work
pJW692	pPB41 with *purR(D203A)* gRNA and repair template	CRISPR recombineering *purR(D203A)*	this work
pJW710	pPB41 with *purR(Y102A)* gRNA and repair template	CRISPR recombineering *purR(Y102A)*	this work
pJW719	pPB41 with *purR(F205A)* gRNA and repair template	CRISPR recombineering *purR(F205A)*	this work
pJW720	pPB41 with *purR(K207A)* gRNA and repair template	CRISPR recombineering *purR(K207A)*	this work
pVL791	pET19-based expression vector with N-terminal HiS7 tag	Expression of His-tagged ORFeome library	PMID: 21876132
pVL847	pET19-based expression vector with N-terminal HiS7-MBP tag	Expression of HisMBP-tagged ORFeome library	PMID: 17178785

Table S5. List of strains.

Strain	Genotype	Reference
JDW2809	*Bacillus subtilis* NCIB3610 *comI (Q12L)*	PMID: 23836866
JDW2231	*Bacillus subtilis* NCIB3610 lacking pBS32 Δ*yjbM* Δ*ywaC relA::mls*	doi: https://doi.org/10.1101/2020.03.22.002139
JDW3359	JDW2809 *purR::ermHI*	this work
JDW3964	JDW2809 *purR(D203A)*	this work
JDW3970	JDW2809 *purR(Y102A/F205A)*	this work
JDW3975	JDW2809 *purR(Y102A/K207A)*	this work
JDW3953	JDW2809 *amyE*::P_{purE}-sfGFP	this work
JDW3951	JDW2231 *amyE*::P_{purE}-sfGFP	this work
JDW3999	JDW3970 *amyE*::P_{purE}-sfGFP	this work
JDW4001	JDW3975 *amyE*::P_{purE}-sfGFP	this work
CF5766	*E. coli* BL21(DE3)/pHM504 (pT7 GppA) Ap	PMID: 23620295
CF7955	*E. coli* BL21(DE3)/pHM1381 (pUM99) (pT7 Rel*Seq*1-385H) Ap	PMID: 23620295
